# Sarcopenia in Hospitalized Patients: A Critical Narrative Review of Diagnostic and Nutritional Management Approaches

**DOI:** 10.3390/nu18193216

**Published:** 2026-09-29

**Authors:** Gavriela Voulgaridou, Spyridoula Ioanna Mourtziapi, George Panoutsopoulos, Paraskevi Detopoulou, Sousana K. Papadopoulou

**Affiliations:** 1 Laboratory of Biological Chemistry, Department of Medicine, School of Health Sciences, Aristotle University of Thessaloniki, 54124 Thessaloniki, Greece; 2 Department of Nutritional Sciences and Dietetics, School of Health Sciences, International Hellenic University, 57400 Thessaloniki, Greece; 3 Department of Nutritional Science and Dietetics, University of Peloponnese, 24100 Kalamata, Greece; i.mourtziapi@hotmail.com (S.I.M.); gpanouts@uop.gr (G.P.); p.detopoulou@go.uop.gr (P.D.)

**Keywords:** sarcopenia, hospitalized patients, nutritional support, oral nutritional supplements, enteral nutrition, parenteral nutrition, muscle wasting, critical illness, anabolic resistance

## Abstract

Sarcopenia is an independent predictor of adverse outcomes in hospitalized patients, associated with increased mortality, prolonged length of stay, and functional decline, yet it remains underdiagnosed owing to inconsistent diagnostic criteria and the practical challenges of assessing acutely ill patients. This narrative review summarizes current evidence on sarcopenia in hospitalized patients, with emphasis on diagnostic approaches, nutritional requirements, and optimal nutritional support strategies. Sarcopenia is particularly prevalent among critically ill patients and is driven by the convergence of systemic inflammation, immobility, and anabolic resistance. Diagnosis remains challenging: no ICU-specific framework exists, and the recent Global Leadership Initiative of Sarcopenia consensus established a unified, setting-independent conceptual definition as a foundation for future operational criteria, with ICU-specific frameworks yet to be developed. Bedside tools, including handgrip strength, bioelectrical impedance analysis, and ultrasound, retain strong prognostic value despite imperfect agreement with reference imaging. Nutritional intervention is a key component of management; however, large-scale trials have demonstrated that higher energy or protein delivery does not universally improve outcomes and may be harmful in selected critically ill patients. A gradual increase in energy and protein delivery according to the phase of illness, with protein targets of ≥1.2–1.5 g/kg/day, is recommended; leucine-enriched supplementation may help counteract anabolic resistance, although direct evidence in non-ICU hospitalized patients remains limited. Early mobilization is an essential complement, as nutrient provision without a mechanical stimulus cannot reverse immobility-induced muscle loss. A practical clinical algorithm integrating screening, nutritional target-setting, route selection, and reassessment is proposed to guide decision-making in this population. Future research should focus on improving the accuracy and feasibility of muscle assessment tools in critically ill patients to enable the timely diagnosis of sarcopenia and nutritional intervention, alongside the development of validated multi-marker panels and adequately powered trials examining the combined effect of nutrition and exercise in this population.

## 1. Introduction

Sarcopenia is a progressive, generalized skeletal muscle disorder characterized by declining muscle mass, muscle strength, and physical performance. The term was first coined by Rosenberg in 1989 to describe age-related muscle loss, but the understanding of the condition has evolved considerably over the past few decades. The revised consensus statement issued by the European Working Group on Sarcopenia in Older People (EWGSOP2) redefined sarcopenia as a disease of muscle failure, placing reduced muscle strength at the center of diagnosis, using low muscle quantity or quality to confirm it, and treating impaired physical performance as an indicator of severity [1]. This shift away from a purely morphological definition toward a functional, more clinically meaningful one reflects a deeper grasp of the condition’s underlying pathophysiology.

Sarcopenia is no longer viewed solely as a syndrome of aging. It is now increasingly recognized as a multifactorial condition that can develop at any age in the presence of acute or chronic illness, immobility, malnutrition, and systemic inflammation, making it particularly relevant among hospitalized patients [1]. Globally, its prevalence is estimated at 9–11% among community-dwelling older adults, rising to 23–24% among hospitalized patients [2], and reaching 37% or higher in hospitalized older adults depending on the diagnostic criteria applied [3]. This considerable heterogeneity in reported prevalence rates reflects the absence of global consensus on diagnostic criteria, as further illustrated by concordance analyses across diagnostic approaches [4].

The clinical significance of sarcopenia in the hospital setting is particularly pronounced. Its presence is associated with an elevated risk of falls, functional decline, prolonged hospitalization, and mortality [5], and it frequently coexists with frailty and malnutrition, giving rise to a complex clinical picture [3]. Despite its clinical relevance, sarcopenia often goes underdiagnosed, as inconsistent diagnostic criteria, the challenges of assessing acutely ill patients, and the lack of standardized evaluation protocols remain significant barriers in clinical practice.

This review aims to summarize current evidence on sarcopenia across the full spectrum of hospitalized adults, from general medical and surgical wards to the intensive care unit, recognizing that the risk of muscle loss and its clinical consequences are shared across these settings, even if their magnitude and management differ. Critically ill patients are treated as a high-risk subgroup within this broader hospitalized population rather than as a separate entity. Specifically, the review addresses diagnostic tools and biomarkers applicable in clinical practice, nutritional requirements, and optimal support strategies across all feeding modalities, oral, enteral, and parenteral. Throughout the review, evidence is presented separately for general ward and ICU populations where available; when data are extrapolated across clinical settings or populations, this is explicitly noted. A practical clinical algorithm integrating the available evidence is proposed to guide nutritional decision-making across hospitalized populations.

## 2. Methods

This narrative review was conducted through a structured, non-systematic literature search using PubMed and Scopus. The search was last updated in June 2026. Search terms combined “sarcopenia”, “muscle mass”, or “muscle wasting” with “hospitalized patients”, “critically ill”, “intensive care unit”, and topic-specific terms including “diagnosis”, “biomarkers”, “nutritional support”, “enteral nutrition”, and “parenteral nutrition”, combined using Boolean operators (AND, OR) across both databases. No language restrictions were applied. Studies were independently screened by two authors based on title, abstract, and full text.

Studies involving adult (≥18 years) hospitalized patients, including general medical/surgical wards, intensive care units, and disease-specific inpatient populations (e.g., oncology, cirrhosis, intestinal failure, postoperative surgical patients), were prioritized. Studies conducted exclusively in community-dwelling, ambulatory, or outpatient populations without a hospitalization component were excluded, unless directly relevant to preoperative optimization preceding hospital admission.

Priority was given to randomized controlled trials, prospective cohort studies, and systematic reviews/meta-analyses published within the last fifteen years, alongside major international consensus guidelines (EWGSOP2, ESPEN, ASPEN). Older seminal studies were retained when they represented foundational evidence or remained the primary reference for a recommendation. Evidence from RCTs and prospective studies was weighted more heavily than observational data; guidelines from ESPEN and ASPEN served as the primary framework for nutritional recommendations. Reference lists of relevant articles were also screened for additional eligible studies.

## 3. Sarcopenia in Hospitalized Patients

Sarcopenia in hospitalized patients presents distinct epidemiological, pathophysiological, and clinical features compared with community-dwelling populations. Hospitalized patients at risk span a broad clinical spectrum, from relatively stable patients in general medical or surgical wards to critically ill patients requiring intensive care. Although the underlying mechanisms of muscle loss are shared across this continuum, their intensity, speed of onset, and clinical consequences differ substantially. The following sections examine the prevalence, underlying mechanisms, and clinical classification of sarcopenia across this spectrum, distinguishing where relevant between the general hospital setting and the intensive care unit (ICU).

### 3.1. Epidemiology

#### 3.1.1. General Hospitalized Patients

The prevalence of sarcopenia among hospitalized patients varies considerably depending on the population studied, the clinical setting, and the diagnostic criteria applied. Comparative analyses using different diagnostic approaches in the same hospitalized cohorts have shown that estimates can diverge substantially depending on the assessment method used, underscoring the lack of a unified diagnostic standard [4]. It is worth emphasizing that hospitalization itself can trigger or worsen de novo sarcopenia: a systematic review found that new-onset sarcopenia developed in 12–38.7% of previously non-sarcopenic older patients following hospital admission, with risk factors including older age, prolonged bed rest, cognitive impairment, and lower baseline body mass index (BMI) [6].

#### 3.1.2. ICU/Critically Ill Patients

Among patients admitted to ICUs, the rates are considerably higher, ranging from 30% to 70% with an overall pooled prevalence of 41% [7]. The presence of sarcopenia in the ICU is associated with a twofold increase in in-hospital mortality risk, as well as increased 30-day and one-year mortality [7]. Ιn septic patients specifically, sarcopenia is associated with significantly prolonged hospital stay (+5.37 days), with a non-significant trend toward longer duration of mechanical ventilatory support [8].

### 3.2. Pathophysiology

#### 3.2.1. General Mechanisms

Sarcopenia in hospitalized patients results from a complex interplay of metabolic, inflammatory, and hormonal mechanisms driving a negative muscle mass balance. Unlike primary age-related sarcopenia, where muscle loss is gradual, this process unfolds rapidly and intensely in the hospital and especially in the ICU setting, driven by the simultaneous activation of multiple catabolic pathways [9].

The systemic inflammatory response is central: pro-inflammatory cytokines such as interleukin-6 (IL-6) and tumor necrosis factor-alpha (TNF-α) activate the ubiquitin-proteasome system while suppressing protein synthesis via mechanistic target of rapamycin complex 1 (mTORC1) inhibition [10].

A further mechanism is anabolic resistance, defined as diminished muscle responsiveness to anabolic stimuli, particularly insulin and amino acids. Chronic inflammation, metabolic dysregulation, and impaired insulin signaling intensify this phenomenon, rendering protein synthesis inefficient even when nutritional intake is adequate [10]. This has direct therapeutic implications, as increasing protein intake alone is insufficient without concurrently addressing underlying inflammation and initiating mobilization.

#### 3.2.2. ICU-Specific Intensification

In critically ill patients, these mechanisms reach their most severe expression. In septic states, pathogen-associated molecular patterns (PAMPs) and damage-associated molecular patterns (DAMPs) further amplify the catabolic response, producing intense muscle breakdown within the first 24–48 h [10]. Immobility-driven losses are faster and more severe when inflammation, sedation, and undernutrition coexist, contributing to ICU-acquired weakness, a distinct clinical syndrome from sarcopenia, though the two frequently coexist in critically ill patients [11]. Additionally, mitochondrial dysfunction and oxidative stress—particularly vulnerable in skeletal muscle during critical illness—directly impair muscle contractility and energy metabolism independent of proteolysis-driven atrophy [12]. Notably, TNF-α-induced reactive oxygen species (ROS) can interfere directly with myofibrillar excitation–contraction coupling, providing a mechanistic link between the inflammatory response described above and impaired muscle function even before measurable muscle loss occurs [13].

### 3.3. Acute Versus Chronic Sarcopenia

Clinically, sarcopenia is classified into chronic and acute forms [1], a distinction with important implications for treatment. Chronic sarcopenia is primarily associated with aging and chronic disease, and is characterized by gradual, progressive muscle loss that develops over months or years. Acute sarcopenia, in contrast, develops over a period of less than six months, typically in response to acute illness, surgery, or trauma, and is characterized by rapid muscle loss and marked functional decline [14,15].

Among hospitalized patients, the acute form predominates. Studies have documented losses of up to 20% in quadriceps cross-sectional area within the first 10 days of hospitalization, with losses occurring faster and more severely in the ICU setting where inflammation, sedation, and immobility coexist [16]. The resulting muscle atrophy can persist for months after discharge, substantially affecting recovery and quality of life [16]. The distinction between acute and chronic sarcopenia is therefore clinically useful, as it informs both urgent nutritional intervention and long-term rehabilitation planning.

### 3.4. Disease-Related Sarcopenia

Sarcopenia in hospitalized patients is frequently classified as secondary, or disease-related sarcopenia, defined as the loss of muscle mass and strength attributable to a specific acute or chronic pathological condition rather than to aging alone [17]. This form occurs across a broad range of clinical conditions, including sepsis [18], trauma, the postoperative period [19], cancer [20], and liver disease [21].

A particularly notable example is cirrhosis, where sarcopenia prevalence can exceed 70% and is associated with markedly increased mortality, with particularly poor outcomes among the subgroup requiring intensive care, in whom sarcopenia emerges as an independent prognostic factor [22]. Similarly, in patients with intestinal failure, prevalence exceeds 70%, driven by malabsorption and intense catabolism, underscoring the critical role of nutritional support as a primary treatment [23].

Unlike primary age-related sarcopenia, disease-related sarcopenia is marked by pronounced catabolic activity and typically coexists with inflammation, metabolic disturbances, and undernutrition, necessitating a comprehensive, multilevel therapeutic approach.

## 4. Diagnostic Approach and Assessment of Sarcopenia in Hospitalized Patients

Diagnosing sarcopenia in hospitalized patients presents a significant clinical challenge. Population heterogeneity, the lack of standardization across diagnostic systems, and the practical limitations of applying functional tests in acutely ill patients all complicate assessment. Nonetheless, early recognition is critical, as delayed diagnosis translates into delayed intervention, with direct consequences for prognosis.

### 4.1. Diagnostic Criteria

Current international frameworks for diagnosing sarcopenia differ in scope and specificity to the hospital setting (Table 1). The EWGSOP2 consensus [1] provides the most widely used reference framework, built on a hierarchical Find-Assess-Confirm-Severity (F-A-C-S) algorithm, and explicitly recognizes an acute, illness- or injury-related form of sarcopenia as distinct from age-related chronic sarcopenia, making it directly applicable, in principle, to hospitalized populations.

Beyond EWGSOP2, several other international frameworks have proposed diagnostic criteria for sarcopenia, including the International Working Group on Sarcopenia [24], the Foundation for the National Institutes of Health Sarcopenia Project [25], and the Asian Working Group for Sarcopenia (AWGS) [26]. These frameworks were developed primarily in community-dwelling populations and do not provide setting-specific algorithms for the hospital context. A partial exception is AWGS 2019 [26], which proposes separate screening pathways for community and hospital settings, recommending calf circumference, SARC-F, or SARC-CalF as first-line screening tools in the hospital context, followed by the assessment of muscle strength and mass to confirm diagnosis. However, its diagnostic thresholds were derived from Asian populations, limiting their direct applicability to Western hospitalized patients.

The most recent and broadest international effort, the Global Leadership Initiative in Sarcopenia (GLIS) Delphi consensus [27], involving 107 experts across 29 countries, explicitly established a unified conceptual definition of sarcopenia, applicable regardless of care setting, age, or underlying condition, as a foundation for future operational criteria. While this represents a landmark step toward global harmonisation, it does not preclude the development of setting-specific operational criteria; however, such criteria, including for the ICU, have yet to be developed, leaving clinicians currently without a dedicated diagnostic framework for critically ill patients.

**Table 1:** Diagnostic frameworks for sarcopenia in hospitalized and ICU patients.

Criteria (Year)	Target Population	Proposed Tools	Diagnostic Criteria/Algorithm
EWGSOP2 (2019) [1]	General framework; explicitly includes acute/hospital-related sarcopenia (<6 months, typically illness- or injury-related) alongside chronic sarcopenia	Handgrip strength or chair-stand test (strength); DXA/BIA/CT/MRI (mass); gait speed/SPPB/TUG (performance)	F-A-C-S algorithm: (1) Find cases: SARC-F; (2) Assess = low strength → probable sarcopenia; (3) Confirm = + low muscle mass; (4) Severity = + low physical performance. Explicitly instructs considering acute illness as a cause of low strength
AWGS (2019) [26]	Community-dwelling and hospitalized older adults (Asian populations)	Calf circumference, SARC-F, SARC-CalF, handgrip strength, 6-m walk test, 5-time chair stand, SPPB, DXA, BIA	Separate algorithms for community and hospital settings. Hospital pathway: screen with CC, SARC-F/SARC-CalF → assess grip strength and muscle mass (DXA/BIA) → diagnose probable sarcopenia or sarcopenia. Severe sarcopenia defined by additional low physical performance.
GLIS (2024) [27]	Global consensus, setting-independent conceptual definition	Muscle mass and muscle strength defined as core “components”; physical performance reclassified as an “outcome,” not a diagnostic component	Agreed that the conceptual definition of sarcopenia should not vary by care setting, age, or condition; unified conceptual definition applicable across all settings; operational criteria for specific contexts, including the ICU, remain to be developed
ANZSSFR Expert Working Group (2022) [3]	Hospitalized older adults (≥65 y), or younger at-risk patients	Grip strength or five-times sit-to-stand; DXA/BIA, or calf circumference as fallback proxy; gait speed for severity	No single screening tool has sufficient evidence; recommends direct strength-based assessment for all hospitalized patients ≥ 65 y, confirmed via mass, severity via gait speed

CC, calf circumference; DXA, dual-energy X-ray absorptiometry; BIA, bioelectrical impedance analysis; CT, computed tomography; MRI, magnetic resonance imaging; SPPB, Short Physical Performance Battery; TUG, Timed Up and Go; SARC-F, Strength, Assistance in walking, Rise from a chair, Climb stairs, Falls questionnaire.

In practice, this leaves clinicians reliant on partial adaptations of general frameworks. The Australian & New Zealand Society for Sarcopenia & Frailty Research (ANZSSFR) Hospital Working Group [3] narrows the gap for general hospital wards, recommending the assessment of probable sarcopenia for all hospitalized patients aged ≥65 years using handgrip strength or the five-times sit-to-stand test as primary strength measures, with calf circumference (<34 cm in men, <33 cm in women) as a practical proxy for muscle mass when dual-energy X-ray absorptiometry (DXA) or bioelectrical impedance analysis (BIA) are unavailable. Confirmation of sarcopenia requires low muscle mass in addition to low strength, while severe sarcopenia is defined by the further presence of impaired physical performance. Importantly, the framework also recommends investigating underlying causes, including acute or chronic disease and malnutrition, in all patients identified with probable sarcopenia or sarcopenia, reflecting the complexity of the hospitalized population. For the ICU specifically, no sarcopenia-specific framework exists; the closest available tool remains the considerably narrower ICU-Acquired Weakness framework [28], a clinically recognized syndrome of generalized, bilateral limb weakness acquired during critical illness, encompassing both neuromuscular dysfunction (critical illness polyneuropathy and myopathy) and diaphragmatic involvement, and typically diagnosed when patients are sufficiently awake to participate in volitional testing. The framework addresses only the functional component via a Medical Research Council (MRC) sum score <48/60, omitting any assessment of muscle mass or quality entirely.

This combination, a setting-independent global definition, a hospital-ward-level adaptation, and a functional-only ICU framework, leaves a genuine methodological gap for critically ill patients.

### 4.2. Assessment Tools in the Hospital Setting

Selecting an appropriate assessment tool in the hospital setting is inherently a trade-off between diagnostic precision and practical feasibility, since the most accurate imaging methods are rarely deployable at the bedside of a critically ill patient. No single tool is therefore sufficient on its own; the strength of assessment lies in combining methods that capture strength, muscle mass, and physical performance according to disease severity, equipment availability, and functional status (Table 2).

#### 4.2.1. Screening Tools and Feasibility

The clinical utility of the SARC-F questionnaire as an initial screening instrument remains a subject of ongoing validation in hospitalized populations. While the tool is practical and widely adopted, its performance in acute inpatient settings is modest: sensitivity and specificity against EWGSOP2-confirmed sarcopenia are both approximately 53%, and its score correlates more closely with reduced strength and physical function than with actual muscle mass loss, limiting its value as a standalone diagnostic instrument [29]. Its negative predictive value of 80.6% has been cited as a potential rule-out metric; however, this should be interpreted with caution given the low sensitivity and the prevalence-dependent nature of NPV. In high-prevalence hospital populations, a negative result may still miss a substantial proportion of true sarcopenia cases. Consistent with the EWGSOP2 algorithm, a negative SARC-F result should therefore prompt rescreening and clinical reassessment rather than definitive exclusion, particularly when clinical suspicion remains [1,29].

When embedded within a sequential diagnostic protocol, however, the picture improves substantially. Step-by-step application of the full EWGSOP2 algorithm yields a confirmed sarcopenia prevalence of 22% in hospitalized older adults, with each successive step adding independent prognostic value for length of stay, fall risk, and quality of life, even after adjustment for age, nutritional status, and comorbidity [30]. The clinical magnitude is evident as median hospitalization was 11 days in non-sarcopenic patients versus 13 days in those with confirmed sarcopenia or dynapenia (*p* < 0.001). Importantly, this 22% figure likely represents an underestimate, as patients excluded from body composition assessment due to clinical complexity were systematically older, more frail, and more malnourished than those included. This confirms that SARC-F combined with handgrip strength offers a clinically meaningful and feasible alternative when advanced body composition testing is unavailable.

In the ICU, screening necessarily incorporates broader clinical constructs. When SARC-F is applied alongside the Clinical Frailty Scale in critically ill elderly patients, sarcopenia prevalence reaches 62.9%, and although severe frailty exerts a stronger independent effect on mechanical ventilation duration and length of stay, sarcopenia itself remains independently associated with a 2.28-fold increase in mortality risk [31].

#### 4.2.2. Strength and Muscle Mass Tools

The following tools are discussed according to the clinical setting in which the available evidence was primarily generated, rather than as setting-exclusive instruments, since many are applicable across both general ward and ICU contexts. Tool selection in practice should be guided by the individual patient’s condition, functional status, and available resources.

##### General Hospital Patients

Handgrip strength assessment stands out among the available tools for its combination of clinical validity, prognostic power, and practical feasibility. In hospitalized older adults, it achieves a completion rate of approximately 95% compared with only 30% for gait speed testing [32]. It is noted that 66% of patients are entirely unable to attempt gait speed testing [32], which in turn gives handgrip strength a feasibility advantage, rendering it a first-line measurement across all ward types. In addition, its prognostic relevance extends to long-term outcomes. For example, in hospitalized adults receiving parenteral nutrition (*n* = 614), both phase angle (OR 0.6, 95% CI 0.5–0.7, *p* < 0.001) and handgrip strength (OR 0.9, 95% CI 0.9–1.0, *p* = 0.036) independently predicted 12-month mortality, with 12-month survival of 57% vs. 84% in patients with low versus normal phase angle [33].

The choice of instrument and diagnostic threshold, however, introduces substantial variability in prevalence estimates. Comparing the Jamar hydraulic dynamometer against the Martin pneumatic vigorimeter across the EWGSOP2, Sarcopenia Definitions and Outcomes Consortium (SDOC), and DO-HEALTH cut-off systems in 376 hospitalized older adults yields probable sarcopenia rates ranging from 68.1% to 89.4%, and confirmed sarcopenia from 39.6% to 50.3%, with older age and lower BMI as the only consistent independent predictors across all models, and sex and fall history non-significant throughout [34]. The vigorimeter offers practical advantages in frail or cognitively impaired patients due to its lower resistance and softer grip surface, and has been used alongside BIA in geriatric inpatients to establish confirmed sarcopenia prevalence of 22.6%, with low calf circumference, low BMI, cognitive impairment, and malnutrition risk emerging as the parameters most consistently associated with the confirmed diagnosis [35]. Its DO-HEALTH-derived cut-offs, however, were derived from community-dwelling populations and require further validation in hospitalized cohorts [35].

Calf circumference has been validated as a rapid bedside proxy for DXA-derived appendicular skeletal muscle mass index in geriatric inpatients, achieving a sensitivity of 71–78% and specificity of 62–79% using sex-specific thresholds, confirming its utility when formal body composition testing is unavailable [36].

It should be noted that bedside ultrasound is not part of the EWGSOP2 diagnostic criteria, and although EWGSOP2 recognises CT as a reference method for muscle quantity, cut-off points for opportunistic CT-derived L3-SMI have not been defined within this framework [1]; both therefore represent complementary tools without a current guideline recommendation for routine sarcopenia diagnosis.

##### ICU Setting

In the ICU, the accuracy of all bedside tools deteriorates due to acute fluid shifts and hemodynamic instability. Computed tomography (CT) imaging at the L3 vertebral level, quantified via the Skeletal Muscle Index (SMI), represents the reference standard for muscle mass assessment in the ICU, valued for its hydration-independence and precision; in septic ICU patients, low CT-derived SMI was present in 23% of cases and independently predicted 1-year mortality (HR 1.9), underscoring its prognostic value even when obtained retrospectively from clinically indicated imaging [37].

BIA systematically overestimates muscle mass relative to CT by approximately 3.35 kg in critically ill surgical patients, with the error amplified in the presence of tissue edema [38], and correlations between bedside methods (BIA, ultrasound, and calf circumference), and CT-derived L3 SMI remain only weak to moderate (r = 0.36–0.62), even as CT identifies reduced muscle mass in 64% of the same patients [39]. Despite this imperfect agreement with reference imaging, specific BIA-derived parameters retain independent prognostic value: phase angle and impedance ratio achieve moderate discrimination for CT-defined low muscularity (AUC 0.67–0.78) and predict ICU length of stay [40], while population-specific predictive equations such as the Talluri formula demonstrate excellent discriminative capacity for low muscle mass (AUC 0.919/0.912 in men/women) despite absolute value disagreement with CT [41].

Bedside ultrasound has emerged as a practical alternative for radiation-free, serial monitoring of muscle architecture throughout ICU admission. Mechanically ventilated patients show quadriceps muscle layer thickness declines of 17–20% over the course of their stay, with impaired diaphragm excursion and lower MRC sum scores independently associated with prolonged ICU length of stay [42], and combined ultrasound and MRC assessment identifies sarcopenia in 36% of ICU patients and independently predicts a 3.82-fold increase in 1-year mortality [43]. In deeply sedated patients where volitional testing is impossible, advanced ultrasound techniques including elastography and microvascular imaging achieve diagnostic accuracy of up to AUC 0.988 for detecting muscle wasting [44]. Despite its practical advantages, bedside ultrasound lacks standardized protocols, limiting inter-operator reproducibility [45].

#### 4.2.3. Physical Performance and Functional Tools

Functional assessment is essential rather than optional, since EWGSOP2 defines severe sarcopenia specifically by impaired physical performance [1]. In the general ward setting, gait-based tests such as the Short Physical Performance Battery (SPPB) and the Timed Up and Go face severe feasibility constraints in acutely ill or immobilized patients, as demonstrated by the 66% non-completion rate for 4-m gait speed compared with approximately 95% for handgrip strength in hospitalized older adults [32]. In such patients, handgrip strength remains the most feasible and clinically meaningful functional assessment.

In the ICU, where gait-based performance tests are rarely feasible, the MRC sum score serves as the primary functional surrogate in patients who are sufficiently awake and cooperative to follow commands, with scores below 48/60 indicating ICU-acquired weakness, a distinct but overlapping clinical construct with sarcopenia [28]. In deeply sedated or delirious patients, the MRC sum score cannot be reliably obtained, and assessment must rely on non-volitional approaches such as muscle ultrasound, BIA-derived phase angle, or CT [37,40,42,44]. The clinical consequences of this functional impairment are profound and well-quantified. Patients classified as weak by MRC criteria show significantly lower rates of live ICU and hospital discharge, incur 30.5% higher total hospital costs, and have substantially higher 1-year mortality compared with non-weak patients (30.6% vs. 17.2%) [46]. Despite this prognostic importance, systematic functional assessment remains under-implemented in routine critical care [47].

Taken together, the evidence converges on a clinically important distinction: while absolute diagnostic agreement between bedside tools and gold-standard imaging frequently deteriorates under the conditions of acute illness, the same bedside parameters retain strong and consistent independent prognostic value. In the general ward, handgrip strength and phase angle perform well within their respective domains; in the ICU, ultrasound and the MRC sum score add critical prognostic information for the critically ill.

**Table 2:** Studies evaluating diagnostic and functional assessment tools across the hospitalized patient spectrum, from general ward to ICU.

Study	Domain	Design/*n*	Country	Age (y)/Sex	Population	Tools Compared	Cut-Off Values	Key Findings
Gade et al., 2020 [29]	Screening	Cross-sectional analysis of RCT data, *n* = 122	Denmark	84.7 ± 6.4; female 80 (65.6%), male 42 (34.4%)	Hospitalized geriatric medical patients ≥ 70 y, mixed conditions	SARC-F validated against both EWGSOP and EWGSOP2 as reference standards	SARC-F ≥ 4; EWGSOP2: HGS <27/< 16 kg, ASMI < 7/<5.5 kg/m^2^	Sarcopenia prevalence with SARC-F: 65.8% (EWGSOP) vs. 21.7% (EWGSOP2) vs. 48.3% at-risk. SARC-F sensitivity 53.8%, specificity 53.2%, NPV 80.6%. SARC-F score correlated with strength and function but not with muscle mass, limiting its diagnostic utility as a standalone screening tool.
Zoico et al., 2026 [30]	Screening algorithm	Prospective observational, *n* = 604	Italy	65–99; men 317 (52.5%), female 287 (47.5%)	Hospitalized older adults ≥ 65 y (Geriatric Department, University Hospital Verona)	EWGSOP2 step-by-step: SARC-F → HGS (Jamar dynamometer) → SMI (BIA, Sergi equation)	SARC-F ≥ 4; HGS < 27/<16 kg; SMI < 7/<5.5 kg/m^2^	Confirmed sarcopenia prevalence 22%; each EWGSOP2 step added prognostic value for LOS, fall risk, QoL; SARC-F + HGS feasible when body composition unavailable
Elmaleh & Mohamed, 2025 [31]	Screening/Clinical outcomes	Prospective cohort, *n* = 70	Egypt	71.3 ± 10.9; male 33 (47.1%), female 37 (52.9%)	Critically ill elderly ICU patients, multiple comorbidities	SARC-F (sarcopenia) + Clinical Frailty Scale (frailty)	SARC-F ≥ 4; CFS 1–9	Sarcopenia in 62.9%; severe frailty in 44.3%; frailty had stronger independent effect on mortality/LOS/MV than sarcopenia; sarcopenia associated with 2.28-fold increased mortality risk
Madrid et al., 2026 [33]	Strength/Mass	Retrospective cohort, *n* = 614	Spain	64 ± 14.6; female 257 (41.8%), male 357 (58.2%)	Hospitalized adults, parenteral nutrition	Phase angle + handgrip strength	Per-unit effect	Phase angle: OR 0.6 (95% CI 0.5–0.7, *p* < 0.001); handgrip: OR 0.9 (95% CI 0.9–1.0, *p* = 0.036) for 12-month mortality; 12-month survival: 57% vs. 84% (low vs. normal phase angle); 62.8% vs. 82.2% (low vs. normal handgrip)
Hanna-Deschamps et al., 2026 [34]	Strength/Mass	Cross-sectional, *n* = 376	Switzerland	82.7 ± 10.9; male 163 (43.4%), female 213 (56.6%)	Hospitalized older adults (acute, rehabilitation, long-term care)	Jamar dynamometer (EWGSOP2, SDOC) vs. Martin vigorimeter (DO-HEALTH1, DO-HEALTH2) + FFMI by BIA	EWGSOP2: <27/<16 kg; SDOC: <35.5/<16 kg; DO-HEALTH: age/sex-specific kPa thresholds; FFMI < 17/<15 kg/m^2^	Probable sarcopenia: 68.1% (Jamar/EWGSOP2), 89.4% (Jamar/SDOC), 72.6% (vigorimeter/DO-HEALTH1), 84.0% (vigorimeter/DO-HEALTH2). Confirmed sarcopenia (low HGS + low FFMI): 39.6–50.3% depending on criteria. Older age and lower BMI were independent predictors across all models; sex and falls were not significant
Darden et al., 2023 [37]	Strength/Mass	Retrospective cohort, *n* = 150	USA	Group 1: 63 (52.69), Group 2: 75 (IQR 63.83); male 87 (58%), female 63 (42%)	Critically ill sepsis patients	CT-derived L3 SMI	<41.6/<32.0 cm^2^/m^2^	Low mass in 23%; independently predicted 1-year mortality (HR 1.9)
Bertschi et al., 2021 [35]	Strength/Mass	Cross-sectional, *n* = 305	Switzerland	84.0 (IQR: 10); male 105 (34.4%), female 200 (65.6%)	Hospitalized geriatric inpatients (acute + rehabilitation)	HGS by pneumatic vigorimeter (DO-HEALTH cut-offs) + ASMI by BIA (EWGSOP2 cut-offs)	HGS < 50/<34 kPa (>75 y), <64/<42 kPa (≤75 y); ASMI < 7/<5.5 kg/m^2^	Probable sarcopenia 24.6%, confirmed 22.6%; low calf circumference, low BMI, cognitive impairment, and malnutrition risk independently associated with confirmed sarcopenia
Kiss, Bertschi et al., 2024 [36]	Strength/Mass	Cross-sectional, *n* = 305	Switzerland	83.5 ± 7; male 105 (34.4%), female 200 (65.6%)	Hospitalized geriatric inpatients	Calf circumference vs. DXA-derived ASMI	CC <31.5 cm (women), <33.5 cm (men)	Sarcopenia prevalence 22.6%; CC identified low ASMI with 78%/71% sensitivity, 79%/62% specificity
Kim et al., 2018 [38]	Strength/Mass	Comparative study, *n* = 135	South Korea	59.3; male 83 (61.5%), female 52 (38.5%)	Critically ill surgical ICU patients	BIA vs. CT-derived muscle mass	Normal SMA > 170/>110 cm^2^	BIA overestimated muscle mass by ~3.35 kg vs. CT, especially with edema
Paulus et al., 2026 [39]	Strength/Mass	Prospective cohort, *n* = 56	Netherlands	70 ± 11; male 39 (69.6%), female 17 (30.4%)	ICU patients, expected stay ≥3 days	BIA, ultrasound, calf circumference vs. CT L3-SMI	New bedside cutoffs proposed	Correlations weak-to-moderate (r = 0.36–0.62); 64% had reduced muscle mass by CT
Kuchnia et al., 2017 [40]	Strength/Mass	Prospective multicenter, *n* = 71	USA/Canada/Netherlands	57 ± 16; male 44 (62%), female 27 (38%)	ICU patients with abdominal CT	BIA phase angle & impedance ratio vs. CT-CSA	Low muscularity: CSA ≤ 170/≤110 cm^2^	Moderate discrimination (AUC 0.67–0.78); predicted ICU length of stay
Looijaard et al., 2019 [41]	Strength/Mass	Prospective observational, *n* = 110	Netherlands	59 ± 17; male 75 (68.2%), female 35 (31.8%)	ICU patients with abdominal CT	3 BIA equations vs. CT muscle mass; phase angle vs. CT density	No fixed BIA cutoff	Absolute values disagreed, but Talluri equation * showed good discrimination (AUC 0.919/0.912)
Ntoumenopoulos et al., 2026 [42]	Strength/Mass	Prospective cohort, *n* = 43	Australia	55 (IQR: 40.5–67.5); male 34 (79%), female 9 (21%)	Mechanically ventilated ICU patients	Serial ultrasound + MRC-SS + ICU Mobility Scale	Measured as relative change	Quadriceps thickness fell 17–20%; diaphragm excursion/MRC-SS associated with ICU LOS
Yanagi et al., 2021 [43]	Strength/Mass + Function	Prospective cohort, *n* = 72	Japan	70 (IQR: 60–76), male 57 (79%), female 15 (21%)	ICU patients, stay ≥ 48 h	Ultrasound quadriceps thickness + MRC sum score	No fixed ultrasound cutoff	ICU-diagnosed sarcopenia in 36%; independently associated with 1-year mortality (HR 3.82)
Ibrahim et al., 2019 [32]	Function	Prospective cohort, *n* = 233	UK	80 (IQR:75–86), Male 139 (60%) Female 94 (40%)	Hospitalized older adults (≥70 y)	Gait speed, grip strength, BIA	—	Only 30% completed gait speed (66% unable); grip strength feasible in 95%; complete EWGSOP2 data available in only 53% of patients; sarcopenia identified in 32% of those fully assessed
Hernández-Socorro et al., 2021 [44]	Function	Prospective observational, *n* = 43	Spain	64.8 ± 14.0, male 25 (58.1), female 18 (41.9)	Long-stay, mechanically ventilated ICU patients	Advanced ultrasound (elastography, microvascular imaging)	—	AUC up to 0.988 for detecting muscle wasting under sedation
Hermans et al., 2014 [46]	Function	Propensity-matched cohort, *n* = 415	Belgium	n/a	Long-stay ICU patients (≥8 days)	MRC sum score	<48 = weak	Weak patients had lower live ICU/hospital discharge, +30.5% costs, higher 1-year mortality (30.6% vs. 17.2%)
Khalil et al., 2023 [47]	Function	Cross-sectional, *n* = 101	Saudi Arabia	67 (IQR: 23); male 52 (51.5%), female 49 (48.5%)	ICU patients	MRC sum score	<48 = weak	ICU-AW incidence 16.8% (*n* = 17); of these, 23.5% significant weakness and 76.5% severe weakness; limited physician awareness identified as primary barrier to systematic assessment

* Talluri equation (total muscle compartment, kg): [0.3 × fat-free mass × log(PA)/0.88] + 0.15 × [(total body water/0.8) × log(PA)], where PA = phase angle. HGS, handgrip strength; ASMI, appendicular skeletal muscle mass index; BIA, bioelectrical impedance analysis; DXA, dual-energy X-ray absorptiometry; CT, computed tomography; L3-SMI, skeletal muscle index at the L3 vertebral level; SMA, skeletal muscle area; CSA, cross-sectional area; MRC-SS, Medical Research Council sum score; ICU-AW, ICU-acquired weakness; ICU, intensive care unit; SPPB, Short Physical Performance Battery; LOS, length of stay; QoL, quality of life; NPV, negative predictive value; CFS, Clinical Frailty Scale; MAC, mid-arm circumference; CC, calf circumference; FFMI, fat-free mass index; AUC, area under the curve. Age values are presented as mean ± standard deviation or median (interquartile range) as reported in the original studies.

## 5. Biomarkers of Sarcopenia

Biomarkers do not serve as standalone diagnostic tools for sarcopenia but rather as reflections of the underlying catabolic and inflammatory processes. No single marker is sufficient to diagnose sarcopenia without combination with imaging or functional assessment. Inflammatory markers, C-reactive protein (CRP) and IL-6 in particular, are the most extensively studied, with sarcopenic patients consistently showing higher levels than non-sarcopenic patients [48,49]. Albumin, despite widespread use, is confounded by inflammation and hydration status and should be interpreted as a marker of morbidity rather than nutritional status [49]. Among the muscle-specific markers, the creatinine-to-cystatin C ratio (Sarcopenia Index) is the most extensively validated, supported by large-cohort evidence [50], though in ICU patients, its accuracy is substantially reduced by catabolism-driven creatinine loss and inflammation-driven cystatin C elevation, requiring cautious interpretation alongside imaging [51].

Several additional markers, such as 3-methylhistidine, myostatin, insulin-like growth factor-1 (IGF-1), testosterone, irisin, and the aspartate aminotransferase/alanine aminotransferase (AST/ALT) ratio, have shown associations with muscle mass or function in the literature, but remain more limited in clinical application, either due to impractical sampling requirements, modest diagnostic accuracy, or a lack of validation specifically in hospitalized populations [48,49,52,53]. Overall, biomarker research in this field remains investigational, and the development of validated, multi-marker panels remains an open research priority, particularly for ICU populations where existing markers are further confounded by the acute inflammatory and catabolic response of critical illness.

## 6. Nutritional Assessment and Management

Sarcopenia in hospitalized patients develops within a setting of increased catabolism, systemic inflammation, and blunted anabolic response, driving a negative muscle protein balance [1]. Nutritional support is a fundamental therapeutic intervention, aimed at preserving or restoring muscle mass, improving functional status, and improving clinical outcomes. Importantly, nutritional intervention is not merely a supportive measure. In sarcopenic patients with sepsis, early enteral feeding has been associated with significantly reduced in-hospital mortality, underscoring its substantive therapeutic role [54].

Nutritional risk should be systematically assessed at admission using validated screening tools: the Nutritional Risk Screening-2002 (NRS-2002) is recommended for general ward patients [55], while the Nutrition Risk in the Critically Ill (NUTRIC) score is preferred in the ICU setting [56] as it incorporates disease severity alongside nutritional parameters.

The nutritional intervention trials with muscle-specific outcomes reviewed in this section are summarized in Table 3.

### 6.1. Energy Requirements

Accurate estimation of energy requirements is a fundamental prerequisite for appropriate nutritional support. Indirect calorimetry is the reference-standard method for estimating energy expenditure, recommended by both the European Society for Clinical Nutrition and Metabolism (ESPEN) [57] and American Society for Parenteral and Enteral Nutrition (ASPEN) [58]. Prior to initiating nutritional support, patients should be assessed for refeeding syndrome risk using validated criteria such as those proposed by ASPEN, which consider factors including prolonged starvation, low BMI, significant weight loss, and electrolyte abnormalities [59]. In patients identified at risk for refeeding syndrome, nutrition should be initiated at an even lower threshold (10–20 kcal/kg/day), with gradual escalation of approximately 33% per day toward the 70% target [59].

In hospitalized polymorbid patients, energy requirements are estimated at 27 kcal/kg/day, with higher targets of 30 kcal/kg/day for severely underweight individuals [60]. In older adults specifically, the ESPEN geriatric guidelines recommend 30 kcal/kg/day as a guiding value, individually adjusted for nutritional status, physical activity, and disease severity [61].

In critically ill patients, ESPEN recommends a target of 20–25 kcal/kg/day when indirect calorimetry is unavailable [57,58], while the 2022 ASPEN guideline suggests 12–25 kcal/kg/day during the first 7–10 days of ICU stay [58], reflecting evidence that higher early energy delivery does not universally improve outcomes. A gradual approach targeting approximately 70% of measured energy expenditure during the acute phase is recommended by both guidelines [62]. However, a gradual approach is equally important in this context: the large multicentre TARGET trial (*n* = 3957), conducted in ICU patients, found that delivering energy-dense versus routine enteral nutrition (EN) produced no difference in 90-day mortality (26.8% vs. 25.7%), suggesting that maximizing caloric delivery alone does not improve survival [63]. In clinical practice, underfeeding remains common as patients consistently receive less energy and protein than they require, particularly during the early days of ICU admission [54].

### 6.2. Protein Requirements

#### 6.2.1. Protein Dosing

Protein is the single most critical nutritional factor in the management of sarcopenia. Adequate protein provision supports muscle protein synthesis, attenuates catabolism, and improves functional outcomes across all hospitalized populations. Targets, however, differ substantially by clinical setting and disease severity.

In hospitalized polymorbid patients, protein targets of >1.0 g/kg/day are recommended, with higher targets of 1.2–1.5 g/kg/day for those who are malnourished or at nutritional risk [60,61]. In hospitalized older adults with sarcopenia specifically, protein should be distributed across at least three meals with ≥25–30 g per meal to maximize muscle protein synthesis in the context of anabolic resistance [64]. During the recovery phase (day 7 onwards), protein targets should be escalated to 1.5–2.0 g/kg/day to support muscle protein synthesis and functional rehabilitation, as resolution of acute inflammation improves anabolic responsiveness [64].

The assumption that higher protein intake universally improves outcomes has not been confirmed in large-scale trials. The EFFORT Protein trial (*n* = 1301), conducted in mechanically ventilated critically ill patients at high nutritional risk, found that high-dose protein (≥2.2 g/kg/day) did not improve time-to-discharge-alive and worsened outcomes in patients with acute kidney injury or high organ failure scores [65].

In critically ill patients, ESPEN recommends 1.3 g/kg/day achieved progressively over the course of ICU stay [57], while ASPEN proposes a broader range of 1.2–2.0 g/kg/day, with the higher end applying to highly catabolic patients [58]. A progressive approach to protein delivery is recommended, with targets increasing gradually over the course of ICU stay [57,58]. In mechanically ventilated ICU patients, a moderate high-protein protocol (1.2 g/kg/day) attenuated quadriceps muscle layer thickness loss compared with standard care (0.75 g/kg/day) [66], and combining protein intake (1.3 g/kg/day) with neuromuscular electrical stimulation and physiotherapy resulted in less muscle volume loss and positive nitrogen balance [67]. In patients with sarcopenic obesity, defined as the coexistence of low muscle mass and excess adiposity, protein should be calculated based on lean body mass to avoid overdosing [62]; in non-sarcopenic obese patients, ESPEN recommends adjusted body weight [ideal BW + (actual BW − ideal BW) × 0.33] [57], while ASPEN proposes a 2–2.5 g/kg ideal body weight [58].

#### 6.2.2. Protein Quality and Anabolic Resistance

Anabolic resistance, the diminished responsiveness of muscle to protein intake, is a central mechanism in hospitalized sarcopenic patients, driven by inflammation, insulin resistance, and hormonal disturbances [14,68]. Protein quality therefore matters as much as quantity. Leucine, the key branched-chain amino acid (BCAA) activating the mTORC1/P70S6K pathway, plays a central role: in older adults, higher leucine doses per meal are required to achieve the same anabolic response as in younger individuals, and a small dose of leucine-enriched essential amino acids (EAAs) (3 g, 40% leucine) can match the protein synthesis response of 20 g whey protein [69]. HMB, a leucine metabolite, stimulates protein synthesis via mTOR while inhibiting proteolysis via the ubiquitin-proteasome system and reduces pro-inflammatory cytokines [50,69]. The most commonly studied dose is 3 g/day [70].

In critically ill patients, high-leucine BCAA supplementation (19 g leucine/day) for 10 days significantly attenuated quadriceps muscle thickness loss compared with the controls (0% vs. −13%, *p* = 0.001), alongside a significant reduction in IL-6 levels (*n* = 40) [71]. A feasibility randomized controlled trial (RCT) testing leucine-enriched EAA versus standard feed in mechanically ventilated trauma patients, however, found that both recruitment and outcome measurement were largely infeasible in this population, highlighting the methodological barriers specific to acute critical illness research [72]. Evidence specifically in non-ICU hospitalized populations remains sparse. Specifically, resistance training combined with hydroxymethylbutyrate (HMB) significantly improved SPPB (+1.32 points, *p* = 0.003), 6MWD (+56.20 m, *p* < 0.001), grip strength (+3.19 kg, *p* = 0.008), ASM (+0.997 kg, *p* = 0.005), and SMI (+0.428 kg/m^2^, *p* = 0.025) and was associated with lower 6-month mortality (OR 0.51, *p* = 0.011), while HMB alone produced only a modest phase angle increase (+0.367, *p* = 0.020) with no effect on other outcomes [73]. In patients with sarcopenia outside the ICU, HMB improves muscle mass and strength [74], whereas it adds no benefit in healthy exercising older adults [70].

It should be noted that direct evidence for leucine-enriched supplementation and HMB in non-ICU hospitalized patients remains sparse. Current recommendations are largely extrapolated from critically ill populations and community-dwelling older adults, and should be applied with appropriate caution in general ward settings.

### 6.3. Micronutrients and Specific Nutritional Substrates

Vitamin D is closely linked to muscle function. Its deficiency is associated with reduced muscle strength, increased fall risk, and accelerated muscle loss. In patients with chronic intestinal failure, high-dose intramuscular vitamin D2 supplementation produced significant improvements in appendicular skeletal muscle mass index, handgrip strength, and gait speed compared with the placebo [75]. The combination of protein and vitamin D appears particularly effective in the hospital setting; for example, in older adults with sarcopenia admitted for physical rehabilitation, a multicomponent oral nutritional supplement (ONS) enriched with whey protein, leucine, and vitamin D administered twice daily for 4–8 weeks resulted in significantly greater improvements in gait speed (*p* < 0.001), muscle mass (*p* < 0.03), handgrip strength, and all functional outcomes (*p* < 0.001) compared with iso-caloric control, alongside shorter rehabilitation duration and length of stay (*p* < 0.001) [76].

Omega-3 fatty acids have anti-inflammatory properties and have been proposed to enhance muscle protein synthesis via inhibition of the nuclear factor kappa B (NF-κB) pathway [77]. However, evidence in hospitalized populations is not supportive. A double-blind RCT in patients undergoing colorectal cancer resection found that perioperative eicosapentaenoic acid (EPA) supplementation (3 g/day for 5 days pre-operatively and 21 days post-operatively) produced no significant difference in lean muscle mass measured by DXA, handgrip strength, or aerobic capacity compared with the placebo [78], consistent with a broader meta-analytic finding of no significant effect of omega-3 supplementation on muscle protein synthesis rates in clinical populations [79].

Glutamine has been proposed as a conditionally essential amino acid during critical illness. However, the large multicentre REDOXS trial, conducted exclusively in critically ill patients with multi-organ failure, found that early glutamine supplementation increased mortality, while antioxidant supplementation had no effect on any outcome [80]. These findings support current guidance against routine high-dose glutamine or antioxidant supplementation in the ICU outside of documented deficiency [49,80]. Evidence in non-ICU hospitalized patients is insufficient to draw conclusions.

**Table 3:** Nutritional intervention studies assessing muscle-specific outcomes in hospitalized patients.

Study	Design/*n*	Age/Sex	Population	Intervention	Outcome Measure	Key Finding
Fetterplace et al., 2018 [66]	Pilot RCT, *n* = 60	n/a	ICU patients	Moderate high-protein (1.2 g/kg/d) volume-based protocol vs. standard (0.75 g/kg/d)	Quadriceps muscle layer thickness (ultrasound)	Muscle loss attenuated by 0.22 cm (95% CI 0.06–0.38); fewer malnourished patients at discharge (7% vs. 28%)
Verceles et al., 2022 [67]	RCT, *n* = 39	n/a	ICU patients under mechanical ventilation	NMES + high protein (1.3 g/kg/d) + physiotherapy vs. standard care	Muscle volume & cross-sectional area (CT)	Less low-extremity muscle volume loss; nitrogen balance turned positive; less delirium
Wandrag et al., 2019 [72]	RCT feasibility, *n* = 8	Control: 60 (28–99), intervention 53 (19–58); male 6 (75%), female 2 (25%)	Trauma patients under mechanical ventilation	Leucine-enriched EAA vs. standard feed	Ultrasound muscle depth (planned)	Recruitment and outcome measurement proved largely infeasible in this population—highlights methodological barriers to this research
Wu et al., 2025 [73]	RCT, *n* = 266	62.0 (IQR: 52.0, 71.0); male 192 (72.2%), female 74 (27.8%)	ICU patients	Resistance training ± HMB vs. standard	Grip strength, ASM, SMI	Resistance training: SPPB +1.32 points (*p* = 0.003), 6MWD +56.20 m (*p* < 0.001), grip strength +3.19 kg (*p* = 0.008), ASM +0.997 kg (*p* = 0.005), SMI +0.428 kg/m^2^ (*p* = 0.025); lower 6-month mortality (OR 0.51, *p* = 0.011); HMB alone: modest phase angle increase only (+0.367, *p* = 0.020)
Gao et al., 2025 [75]	RCT, *n* = 84	Control: 52.8 ± 15.6, intervention: 52.9 ± 16.2; male 47 (56%), female 37 (44%)	Chronic intestinal failure patients	High-dose vitamin D2 (IM) vs. placebo	ASMI, handgrip, gait speed	Significant improvement in ASMI, handgrip, and gait speed (*p* < 0.05 for all)
Wulandari et al., 2025 [71]	Multicentre double-blind RCT, *n* = 40	49 (IQR: 21); male 31 (77.5%), female 9 (22.5%)	ICU patients	High-leucine BCAA (19 g leucine/day) enterally/parenterally vs. control, 10 days	Quadriceps femoris muscle thickness (ultrasound)	Significantly less muscle thickness loss in BCAA group (0% vs. −13%, *p* = 0.001); significant reduction in IL-6
Rondanelli et al., 2020 [76]	Double-blind RCT, *n* = 140	81 ± 6; male 52 (37%), female 88 (63%)	Hospitalized older adults with sarcopenia admitted for physical rehabilitation	Whey protein + leucine + vitamin D enriched formula vs. iso-caloric control, twice daily, 4–8 weeks	Gait speed, muscle mass, handgrip strength, SPPB, functional status, LOS	Significantly greater improvement in gait speed (*p* < 0.001), muscle mass (*p* < 0.03), and all functional outcomes (*p* < 0.001); shorter rehabilitation (*p* < 0.001) and hospital stay (*p* < 0.001)
Hossain et al., 2019 [78]	Double-blind RCT, *n* = 61	68.3 ± 0.95; male 42 (69%), female 19 (31%),	Hospitalized patients undergoing colorectal cancer resection	EPA 3 g/day vs. placebo, 5 days pre-op + 21 days post-op	Lean muscle mass, handgrip strength, aerobic capacity (CPET)	No significant difference in LMM (MD 704.77 g; 95% CI −1045.6 to 2455.13 g; *p* = 0.42), aerobic capacity (*p* = 0.18), or handgrip strength between groups

ONS, oral nutritional supplements; HMB, β-hydroxy-β-methylbutyrate; BCAA, branched-chain amino acids; EAA, essential amino acids; BIA, bioelectrical impedance analysis; DXA, dual-energy X-ray absorptiometry; NMES, neuromuscular electrical stimulation; ICU, intensive care unit; ASMI, appendicular skeletal muscle mass index; SMI, skeletal muscle index; LOS, length of stay; SPPB = Short Physical Performance Battery; EPA = Eicosapentaenoic Acid; CPET = Cardiopulmonary Exercise Testing. Age values are presented as mean ± standard deviation or median (interquartile range) as reported in the original studies.

## 7. Nutritional Support Strategies in Hospitalized Patients

**Figure 1:**
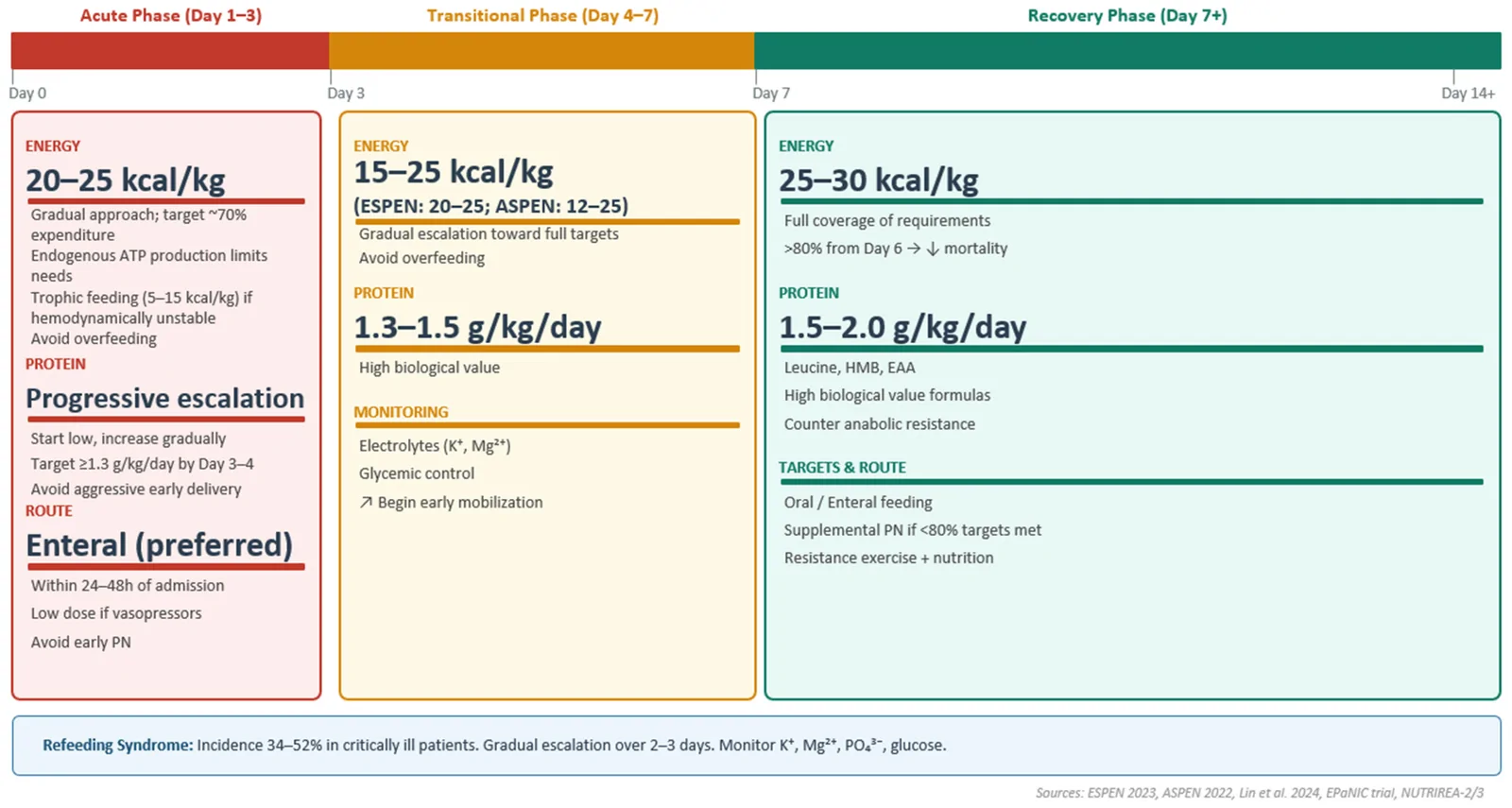
Phases of nutritional support in critically ill patients. Phases of nutritional support across the continuum of critical illness. Energy and protein targets are adapted from the ESPEN and ASPEN guidelines. Trophic feeding (5–15 kcal/kg/day) applies to hemodynamically unstable patients during the acute phase [57,58,81,82,83,84]. Abbreviations: EN, enteral nutrition; PN, parenteral nutrition; HMB, β-hydroxy-β-methylbutyrate; EAA, essential amino acids.

Nutritional support in hospitalized patients with sarcopenia follows a hierarchical, individualized approach, guided primarily by gastrointestinal function, disease severity, and the patient’s ability to meet their nutritional needs orally (Table 5). Timely initiation of the appropriate feeding modality is critical, as delays are associated with worsening muscle loss and poorer prognosis. In sarcopenic patients with acute illness, and particularly in sepsis, early enteral feeding has been shown to significantly reduce in-hospital mortality, underscoring the therapeutic role of nutrition beyond mere substrate provision [54]. The comprehensive nutritional strategy across phases of critical illness is illustrated in Figure 1.

### 7.1. Oral Nutrition

When feasible, oral feeding is the first and preferred option. However, in hospitalized patients with sarcopenia, standard diet alone is often insufficient to meet the increased nutritional needs owing to anorexia, fatigue, dysphagia, or anabolic resistance. A stepwise escalation is therefore applied with dietary fortification through increased caloric and protein density in small frequent meals. When intake remains persistently below 60–70% of the estimated requirements, ONS should be considered, taking into account the expected duration of insufficient intake, swallowing safety, gastrointestinal function, and overall clinical condition [61]. High-protein ONS, particularly those enriched with leucine, HMB, or vitamin D, represent the most evidence-supported intervention in this context [85,86].

A randomized controlled trial in inpatients across acute care and convalescent rehabilitation wards found that a branched-chain amino acid (BCAA)- and vitamin D-enriched ONS administered twice daily until discharge significantly increased SMI compared with standard care (+4.3% vs. −2.2%, *p* = 0.014), despite showing no difference in activities of daily living [85]. A large multicentre double-blind RCT (NOURISH, *n* = 722) in malnourished hospitalized older adults (≥65 y) found that a high-protein ONS enriched with HMB, initiated at admission and continued for 90 days post-discharge, significantly reduced the 90-day mortality (*p* < 0.05) and attenuated lean mass loss compared with the placebo, although the primary composite endpoint of readmission or death did not reach statistical significance [86]. Notably, both trials demonstrate a recurring pattern in this literature: nutritional intervention may preserve or increase muscle mass without a parallel gain in functional outcomes, reinforcing the rationale for combining ONS with structured mobilization wherever feasible.

### 7.2. Enteral Nutrition

#### 7.2.1. General Principles

When oral feeding is insufficient or unsafe but the gastrointestinal tract remains functional, enteral nutrition is the method of choice. Beyond its nutritional value, enteral feeding provides important non-nutritional benefits such as preserving intestinal barrier integrity, reducing bacterial translocation, maintaining gut microbial diversity, and supporting immune function [57,58]. Compared with parenteral nutrition, enteral feeding is associated with a significantly lower risk of infectious complications [57]. A stepwise approach is recommended as enteral feeding is initiated when oral intake fails to meet the nutritional targets despite dietary optimization and ONS.

#### 7.2.2. ICU Setting

Both ESPEN and ASPEN recommend initiating enteral feeding within 24–48 h of ICU admission in critically ill patients not expected to resume oral intake [57,58]. In patients with sarcopenia and sepsis, early enteral feeding has demonstrated substantial benefits including reduced mortality and shorter hospital stay [54]. However, in patients receiving high-dose vasoactive agents, enteral feeding at full caloric targets has been associated with an increased risk of intestinal ischemia, as demonstrated in the NUTRIREA-2 and NUTRIREA-3 trials; in these cases, low-dose trophic feeding (5–15 kcal/kg/day) is recommended until hemodynamic stability is achieved [81,82]. The risk of refeeding syndrome, with a reported incidence of 34–52% among critically ill patients, further requires gradual nutrient escalation over 2–3 days with close electrolyte monitoring [87].

For critically ill patients with sarcopenia, high-protein calorically dense formulas are preferred. A randomized trial in mechanically ventilated ICU patients with severe pneumonia comparing high-protein (1.8 g/kg/day) with standard-protein (1.2 g/kg/day) enteral formulas demonstrated significantly less quadriceps atrophy in the high-protein group by day 10 (13.97% vs. 18.96%, *p* < 0.001) via serial ultrasound, although diaphragmatic thickness declined similarly in both groups [88]. An emerging area concerns the modulation of the gut–muscle axis through synbiotic-supplemented enteral nutrition. A triple-blind RCT in critically ill ICU patients found that EN supplemented with synbiotics (*Lactobacillus*, *Bifidobacterium*, *Streptococcus*, fructooligosaccharides) maintained mid-arm circumference (MAC) and estimated muscle mass over 14 days, while the placebo group showed significant MAC reduction (*p* < 0.001) and approximately 2 kg muscle mass decrease, though between-group differences did not reach statistical significance [89].

### 7.3. Parenteral Nutrition

#### 7.3.1. General Principles

Parenteral nutrition (PN) is indicated when enteral feeding is not possible or insufficient, for example, in cases of ileus, severe malabsorption, intestinal failure, or gastrointestinal complications [57,62]. In patients with chronic intestinal failure and sarcopenia, long-term home parenteral nutrition has been associated with documented longitudinal increases in skeletal muscle mass at the L3 vertebral level via abdominal CT [90]. Clinicians should also account for differences in macronutrient caloric density between enteral and parenteral nutrition, as dextrose provides 3.4 kcal/g compared with 4 kcal/g for enteral carbohydrates, and lipid emulsions provide 1.1 kcal/mL (10% solution) or 2.0 kcal/mL (20% solution) [58] (Table 4).

#### 7.3.2. ICU Setting

The timing of parenteral nutrition initiation is critically important. The EPaNIC trial (*n* = 4640) demonstrated that early supplemental parenteral nutrition within 48 h in the ICU was associated with prolonged organ support dependence, increased infections, and higher incidence of ICU-acquired weakness compared with delayed initiation [83]. A propensity-matched subanalysis (*n* = 415) quantified the muscle-specific consequence: fewer patients developed ICU-acquired weakness with late versus early PN (8.0% absolute difference, *p* < 0.0001), and muscle biopsies showed greater autophagy activation with late PN, suggesting improved myofiber quality control [46].

The effect of protein dose delivered via enteral and/or parenteral nutrition was further examined in a single-blind RCT in mechanically ventilated ICU patients (*n* = 42), comparing high protein (1.8 g/kg/day) versus standard protein (1.2 g/kg/day) with energy individualized via indirect calorimetry: both groups lost quadriceps muscle mass over 4 weeks with no significant between-group difference (control: −0.28 ± 0.08 mm vs. intervention: −0.15 ± 0.08 mm; *p* = 0.368), reinforcing that protein dose alone does not prevent muscle atrophy in the acute phase [91]. Furthermore, propofol, widely used for sedation in ICU patients, is formulated as a 10% lipid emulsion and contributes 1.1 kcal/mL in non-nutritive calories that must be factored into the total energy calculations to avoid unintentional overfeeding [92]. Parenteral nutrition in the ICU is therefore best applied when enteral feeding is fully contraindicated, when severe malnutrition is documented at admission, or when enteral feeding fails to meet the targets after the first week [62].

### 7.4. Combined Enteral–Parenteral Nutrition

#### 7.4.1. General Principles

In cases where enteral feeding alone is insufficient to meet the nutritional targets without being contraindicated, supplemental parenteral nutrition is applied. The decision to initiate supplemental parenteral nutrition (SPN) should be individualized, taking into account nutritional status at admission, degree of energy deficit, gastrointestinal tolerance, and clinical trajectory, rather than applied as a universal threshold [62].

#### 7.4.2. ICU Setting

In critically ill patients with pre-existing malnutrition or low BMI, SPN initiated after the first week in the ICU can prevent cumulative energy deficits and improve outcomes [62]. Data from a large international database (*n* = 3544) showed that energy intake below 60% of requirements was associated with significantly increased 28-day mortality, whereas achieving over 80% coverage by day 6 was associated with reduced mortality, though these thresholds should be interpreted as clinical guidance rather than rigid targets, given the heterogeneity of the ICU population [84]. A pilot RCT [93] testing combined SPN and EN in underweight and obese ICU patients with acute respiratory failure found non-significant but consistent trends toward improved mortality and functional outcomes with no increase in infectious risk. Implementation of combined feeding always requires monitoring for refeeding syndrome, hyperglycemia, and electrolyte disturbances.

Data specifically examining the impact of combined EN+PN on sarcopenia-related parameters such as muscle mass or strength are currently lacking, representing an important gap for future research.

### 7.5. ICU-Specific Considerations

Nutritional strategy in the ICU depends on the phase of illness. During the acute phase (days 1–3), endogenous energy production from catabolism is substantial and targeting full caloric coverage may be harmful. In hemodynamically unstable patients, particularly those receiving high-dose vasoactive agents, low-dose trophic feeding (5–15 kcal/kg/day) is preferred until stabilization [81,82], whereas in hemodynamically stable patients, hypocaloric feeding (not exceeding 70% of estimated energy expenditure) with progressive advancement is recommended [57]. Protein delivery should follow a progressive approach during this phase, with gradual escalation toward target rather than immediate full-dose provision, to avoid the potential adverse effects of early aggressive protein delivery in the most critically ill [57,58,62]. In the transitional and recovery phases (day 4–7 onward), gradually increasing targets to exceed 80% of energy requirements is associated with reduced mortality [94]. Early mobilization is a necessary complement throughout, as nutrient provision without a corresponding mechanical stimulus cannot reverse immobility-induced muscle loss [95].

Sarcopenic obesity presents a particular challenge: apparently adequate muscle mass is masked by excess adiposity, and energy targets should be based on ideal or adjusted body weight with protein calculated from lean body mass [58]. Current recommendations in this subgroup are largely extrapolated from general critical care nutrition principles rather than dedicated trials.

**Table 5:** Nutritional intervention studies assessing muscle-specific outcomes in hospitalized and critically ill patients.

Study	Design/*n*	Age/Sex	Population	Route/Timing	Intervention	Muscle Outcome Measure	Key Finding
Moriwaki et al., 2019 [85]	RCT, *n* = 55	Intervention: 74.8 ± 12, control group: 77.7 ± 13.2; male 30 (54.5%), female 25 (45.5%),	Inpatients, acute + rehab wards	Oral (ONS)	BCAA + vitD supplement vs. control	SMI	+4.3% vs. −2.2% (*p* = 0.014); no ADL difference
Deutz et al., 2016 [86]	Multicenter RCT, *n* = 722	Intervention: 77.7 ± 8.2, control group: 78.1 ± 8.6; male 298 (41.3%), female 424 (58.7%)	Hospitalized older adults (≥65 y) with malnutrition risk	Oral (ONS)/Initiated at admission up to 90 days post-discharge	High-protein ONS enriched with HMB vs. placebo	Body weight, lean body mass (BIA), handgrip strength	No significant difference in primary composite endpoint (readmission or death); HP-HMB associated with 50% reduction in 90-day post-discharge mortality (*p* < 0.05) and attenuation of lean mass loss; improved nutritional status
Liu et al., 2024 [88]	RCT, *n* = 120	Intervention: 61.15 ± 11.95, standard of care group: 61.70 ± 12.17, male 80 (66.7%), female 40 (33.3%)	ICU, severe pneumonia, ventilated	Enteral	High (1.8 g/kg) vs. standard (1.2 g/kg) protein	Quadriceps/diaphragm thickness (ultrasound)	Significantly less quadriceps atrophy with high-protein vs. standard-protein enteral formula (13.97% vs. 18.96% loss, *p* < 0.001)
Hermans et al., 2014 [46]	Propensity-matched analysis, *n* = 415 (EPaNIC)–not a RCT	n/a	Long-stay ICU	Parenteral (timing)	Early vs. late PN	MRC score (ICU-AW) + muscle biopsies	Fewer patients developed ICU-AW with late vs. early PN (8.0% absolute difference, *p* < 0.0001); muscle biopsies showed greater autophagy activation with late PN, suggesting improved myofiber quality control.
Dresen et al., 2021 [91]	Single-blind RCT, *n* = 42	65 ± 15; male 30 (71.4%), female 12 (28.6%)	Critically ill ICU patients, mechanically ventilated (Germany)	Enteral/Parenteral	High protein (1.8 g/kg/day) vs. standard (1.2 g/kg/day) enteral/parenteral nutrition; energy individualized via indirect calorimetry	Quadriceps muscle layer thickness (ultrasound), serial measurements over 4 weeks	Both groups lost quadriceps muscle mass over 4 weeks; no significant between-group difference in QMLT change (control: −0.28 ± 0.08 mm vs. intervention: −0.15 ± 0.08 mm; *p* = 0.368)
Seifi et al., 2022 [89]	Triple-blind RCT, *n* = 38	Intervention: 38.5 ± 17.94, control group: 47.61 ± 22.51, male 23 (60.5%), female 15 (39.5%)	Critically ill ICU patients requiring EN (Iran)	Enteral nutrition; initiated within 24–48 h of ICU admission	EN + synbiotic (*Lactobacillus*, *Bifidobacterium*, *Streptococcus*, FOS) vs. EN + placebo, up to 14 days	Mid-arm circumference (MAC), 24-h urine creatinine, creatinine-height index, estimated muscle mass	Synbiotic group maintained MAC and muscle mass while placebo group showed significant MAC reduction (*p* < 0.001) and ~2 kg muscle mass decrease; differences between groups not statistically significant on day 14
Casaer et al., 2011 [83]	RCT, *n* = 4640	Late PN 64 ± 15, early PN 64 ± 14; male 2972 (64.3%), female 1668 (35.7%)	Critically ill ICU patients (mixed medical/surgical), Belgium	Parenteral (timing)	Early PN (day 2) vs. late PN (day 8)	ICU/hospital LOS, infections, mortality, ICU-AW	Late PN: shorter ICU stay (median 3 vs. 4 days, *p* = 0.02), faster weaning from MV (−9.7%, *p* = 0.006), fewer infections (22.8% vs. 26.2%, *p* = 0.008), shorter renal replacement therapy (−3 days, *p* = 0.008); no difference in ICU, hospital, or 90-day mortality

ONS, oral nutritional supplements; EN, enteral nutrition; PN, parenteral nutrition; SPN, supplemental parenteral nutrition; HMB, β-hydroxy-β-methylbutyrate; BCAA, branched-chain amino acids; EAA, essential amino acids; FOS, fructooligosaccharides; MAC, mid-arm circumference; ASM, appendicular skeletal mass; SMI, skeletal muscle index; ICU, intensive care unit; ICU-AW, ICU-acquired weakness; MRC, Medical Research Council; ADL, activities of daily living; LOS, length of stay; DXA, dual-energy X-ray absorptiometry; CPET, cardiopulmonary exercise testing. Age values are presented as mean ± standard deviation or median (interquartile range) as reported in the original studies.

## 8. Clinical Algorithm

**Figure 2:**
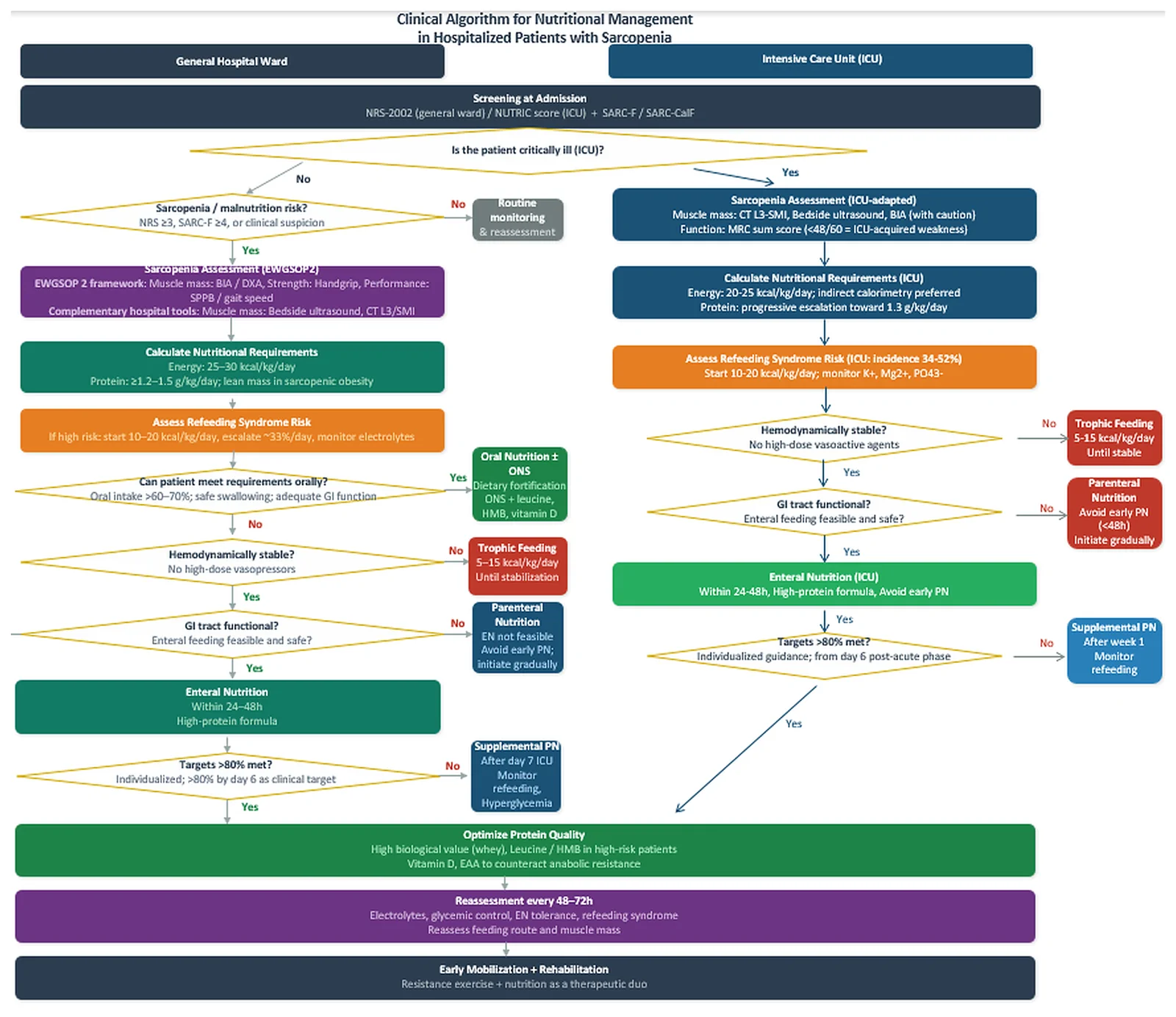
Clinical algorithm for nutritional management in hospitalized sarcopenic patients. Stepwise clinical algorithm for nutritional decision-making in hospitalized patients with diagnosed or suspected sarcopenia. Route selection is guided by hemodynamic stability and gastrointestinal function. Separate pathways are provided for general hospital ward and ICU settings, reflecting differences in evidence base and clinical context. The left pathway (grey arrows) refers to general hospital wards and the right pathway (dark blue arrows) to the ICU setting. Diamonds indicate decision points (green, yes; red, no). Box colors denote assessment and reassessment (purple), nutritional requirements and primary nutritional interventions (green), refeeding syndrome risk assessment (orange), feeding strategies requiring caution (red), supplemental parenteral nutrition (blue), and rehabilitation (dark blue). Recommendations are based on the EWGSOP2 consensus and sarcopenia screening recommendations [1,3], the diagnostic framework for ICU-acquired weakness [28], ESPEN and ASPEN guidelines [57,58], and supporting evidence on refeeding syndrome [60,61], oral intake and nutritional supplementation [62,76,81,86], trophic enteral feeding [81,82], parenteral nutrition [84,91], and early mobilization [95]. Abbreviations: BIA, bioelectrical impedance analysis; CT, computed tomography; SMI, skeletal muscle index; SPPB, Short Physical Performance Battery; ONS, oral nutritional supplements; EN, enteral nutrition; PN, parenteral nutrition; HMB, β-hydroxy-β-methylbutyrate; EAA, essential amino acids; ICU, intensive care unit. Note: This algorithm is intended as a practical clinical guidance tool, integrating available evidence with expert consensus where direct evidence is limited or population-specific. No equivalent validated algorithm currently exists for the full spectrum of hospitalized patients with sarcopenia. Recommendation strength varies across steps; clinical judgment and individual patient assessment should always guide the final decisions.

Translating scientific knowledge into clinical practice requires a structured approach that guides the clinician from the initial identification of sarcopenia through nutritional target-setting, route selection, and ongoing monitoring. The algorithm presented here (Figure 2) integrates the ESPEN and ASPEN recommendations with the evidence presented in the preceding sections and is applicable to all hospitalized patients with diagnosed or suspected sarcopenia, from general medical and surgical wards to the ICU, with ICU-specific adaptations indicated where recommendations differ [57,58].

At admission, all patients should undergo nutritional risk screening using the NRS-2002 in general wards or the NUTRIC score in the ICU, combined with sarcopenia screening using SARC-F or SARC-CalF [1,3]. Where feasible, diagnosis should be confirmed according to the EWGSOP2 criteria, using muscle strength (handgrip dynamometry) and muscle mass (DXA or BIA), with physical performance used to grade severity [1,3]. When these measures cannot be applied, particularly in the ICU, phase angle, bedside ultrasound, or CT-derived L3-SMI from clinically indicated imaging may support risk stratification and monitoring, but should not be considered formal diagnostic confirmation [37,40,42,43]. This initial evaluation establishes the baseline and informs the urgency of nutritional intervention. Nutritional requirements are then calculated, targeting an energy intake of 20–25 kcal/kg/day [57] or 12–25 kcal/kg/day [58] during the acute phase, progressing to 25–30 kcal/kg/day during recovery [57], and protein intake of ≥1.2–1.5 g/kg/day, calculated from the lean body mass in patients with sarcopenic obesity [57,58,64]. Indirect calorimetry is recommended where available, particularly in ICU patients [57,58].

Before selecting a feeding route, refeeding syndrome risk should be assessed in all patients using the ASPEN criteria [59]; in those identified as high risk, nutritional support should be initiated at 10–20 kcal/kg/day with gradual escalation of approximately 33% per day toward the target, alongside close electrolyte monitoring. The clinician must then determine whether the patient can meet their nutritional requirements orally. If oral intake exceeds 60–70% of the calculated requirements and swallowing is safe [61], oral feeding with dietary fortification is the first-line approach, escalating to ONS enriched with leucine, HMB, or vitamin D when intake remains insufficient [76,86]. When oral intake falls below this threshold, taking into account the expected duration of insufficient intake, swallowing safety, gastrointestinal function, and overall clinical condition, escalation to artificial nutrition should be considered [57]. Route selection is then guided by two sequential questions: hemodynamic stability and gastrointestinal function. In hemodynamically unstable patients receiving high-dose vasoactive agents, low-dose trophic enteral feeding (5–15 kcal/kg/day) is preferred until stability is achieved [81,82]. In stable patients with a functional gastrointestinal tract, enteral nutrition is the method of choice, initiated within 24–48 h [57,58]. When enteral feeding is contraindicated or fails to meet the targets after the first week, parenteral nutrition, initiated gradually and with strict avoidance of overfeeding, is indicated [57,83]. In patients with pre-existing severe malnutrition where enteral feeding alone is insufficient, supplemental parenteral nutrition may be added after the first week [57,58]. When enteral feeding consistently fails to achieve more than 80% of the calculated energy and protein targets by day 6 of the post-acute phase, supplemental parenteral nutrition should be considered to prevent cumulative nutritional deficits [57,59,84].

Regardless of feeding route, nutritional tolerance, electrolytes, glycemic control, and target achievement should be systematically reassessed every 48–72 h [57], with targets adjusted according to the phase of illness. Early mobilization remains an integral complement throughout all phases [95], as nutritional provision without a mechanical stimulus cannot reverse immobility-induced muscle loss.

## 9. Limitations

The present review has several limitations that should be considered when interpreting its findings. First, the non-systematic nature of the literature search, while appropriate for a narrative review, introduces the possibility of selection bias and limits reproducibility. Second, the search was conducted across two databases only (PubMed and Scopus), and relevant studies indexed exclusively in other sources may have been missed. Third, the included studies are characterized by substantial heterogeneity in population characteristics, clinical settings, and diagnostic definitions of sarcopenia, which limits direct comparability across findings. Fourth, evidence is frequently extrapolated across different hospital settings, from general medical and surgical wards to the intensive care unit, and the applicability of ICU-derived findings to broader hospitalized populations, and vice versa, cannot always be assumed. Fifth, direct evidence in non-ICU hospitalized patients with sarcopenia remains sparse for several nutritional interventions, and current recommendations in this population are largely informed by critically ill patient data or community-dwelling older adults. These limitations should be considered when translating the findings of this review into clinical practice.

## 10. Conclusions

Sarcopenia in the hospital setting is an independent predictor of adverse outcomes, associated with increased mortality, prolonged hospitalization, and functional decline, with particularly high prevalence among critically ill patients. These findings support the need for systematic screening at admission rather than reserving assessment for cases of overt malnutrition. Diagnostic tools such as bedside ultrasound, phase angle, and CT-derived SMI have demonstrated prognostic value in hospitalized populations and represent valuable complementary tools, particularly when formal diagnostic measures cannot be applied. However, a significant diagnostic gap persists in the ICU setting: the GLIS consensus established a unified conceptual definition applicable across all settings as a foundation for future operational criteria, but setting-specific frameworks, including one for the ICU, remain to be developed.

Regarding nutritional intervention, current evidence indicates that higher energy or protein delivery does not universally improve outcomes and may be harmful in selected critically ill patients. Gradual escalation guided by phase of illness and strict avoidance of early overfeeding remain the cornerstones of nutritional management. Protein provision with emphasis on leucine-enriched sources to overcome anabolic resistance is supported primarily by evidence from critically ill and community-dwelling older adult populations, with direct evidence in non-ICU hospitalized patients remaining limited. HMB supplementation shows heterogeneous effectiveness, with greater benefit observed in catabolic states. Critically, nutritional support cannot be viewed in isolation; early mobilization is an indispensable complement, as nutrient provision without a mechanical stimulus cannot reverse immobility-induced muscle loss.

Future research should focus on improving the accuracy and feasibility of muscle assessment tools in critically ill patients to enable the timely diagnosis of sarcopenia and nutritional intervention, alongside the development of validated multi-marker panels and adequately powered trials examining the combined effect of nutrition and exercise in this population.

## Figures and Tables

**Table 4:** Macronutrient caloric density in enteral and parenteral nutrition.

Macronutrient	Enteral (EN)	Parenteral (PN)
Protein/Amino acids	4 kcal/g	4 kcal/g
Carbohydrates	4 kcal/g	3.4 kcal/g (dextrose monohydrate)
Lipids	9 kcal/g	1.1 kcal/mL (10% emulsion)/2.0 kcal/mL (20% emulsion)
Propofol	-	1.1 kcal/mL (10% lipid emulsion)

## Data Availability

No new data were created or analyzed in this study. Data sharing is not applicable to this article.

## References

[1] Cruz-Jentoft A.J., Bahat G., Bauer J., Boirie Y., Bruyère O., Cederholm T., Cooper C., Landi F., Rolland Y., Sayer A.A. (2019). Sarcopenia: Revised European consensus on definition and diagnosis. Age Ageing.

[2] Papadopoulou S.K., Tsintavis P., Potsaki G., Papandreou D. (2020). Differences in the Prevalence of Sarcopenia in Community-Dwelling, Nursing Home and Hospitalized Individuals. A Systematic Review and Meta-Analysis. J. Nutr. Health Aging.

[3] Daly R.M., Iuliano S., Fyfe J.J., Scott D., Kirk B., Thompson M.Q., Dent E., Fetterplace K., Wright O.R.L., Lynch G.S. (2022). Screening, Diagnosis and Management of Sarcopenia and Frailty in Hospitalized Older Adults: Recommendations from the Australian and New Zealand Society for Sarcopenia and Frailty Research (ANZSSFR) Expert Working Group. J. Nutr. Health Aging.

[4] Venegas-Sanabria L.C., Borda M.G., Murcia-Soriano L.F., Ramos-Caballero D.M., Tordecilla-Sanders A., Garcia-Laguna G., Vargas-Pinilla O. (2024). Unveiling a hidden burden: Exploring sarcopenia in hospitalized older patients through concordance and cluster analysis. BMC Geriatr..

[5] Beaudart C., Zaaria M., Pasleau F., Reginster J.-Y., Bruyère O. (2017). Health Outcomes of Sarcopenia: A Systematic Review and Meta-Analysis. PLoS ONE.

[6] Wan S.N., Thiam C.N., Ang Q.X., Engkasan J., Ong T. (2023). Incident sarcopenia in hospitalized older people: A systematic review. PLoS ONE.

[7] Zhang X.-M., Chen D., Xie X.-H., Zhang J.-E., Zeng Y., Cheng A.S. (2021). Sarcopenia as a predictor of mortality among the critically ill in an intensive care unit: A systematic review and meta-analysis. BMC Geriatr..

[8] Ni F., Zheng L. (2025). Impact of sarcopenia and frailty on outcomes of patients with sepsis or septic shock: A systematic review and meta-analysis. Front. Nutr..

[9] Van Der Steen-Dieperink M.J.M.M., Koekkoek W.A.C., Kouw I.W.K. (2025). Sarcopenia and frailty in critical illness. Curr. Opin. Clin. Nutr. Metab. Care.

[10] Schefold J.C., Wollersheim T., Grunow J.J., Luedi M.M., Z’Graggen W.J., Weber-Carstens S. (2020). Muscular weakness and muscle wasting in the critically ill. J. Cachexia Sarcopenia Muscle.

[11] Kortebein P., Ferrando A., Lombeida J., Wolfe R., Evans W.J. (2007). Effect of 10 days of bed rest on skeletal muscle in healthy older adults. JAMA.

[12] Klawitter F., Ehler J., Bajorat R., Patejdl R. (2023). Mitochondrial Dysfunction in Intensive Care Unit-Acquired Weakness and Critical Illness Myopathy: A Narrative Review. Int. J. Mol. Sci..

[13] Batt J., Mathur S., Katzberg H.D. (2017). Mechanism of ICU-acquired weakness: Muscle contractility in critical illness. Intensive Care Med..

[14] Welch C., Bravo L., Gkoutos G., Greig C., Lewis D., Lord J., Majid Z., Masud T., McGee K., Moorey H. (2024). Establishing Predictors of Acute Sarcopenia: A Proof-Of-Concept Study Utilising Network Analysis. Aging Dis..

[15] Montero-Errasquín B., Cruz-Jentoft A.J. (2023). Acute Sarcopenia. Gerontology.

[16] van Gassel R.J.J., Baggerman M.R., van de Poll M.C.G. (2020). Metabolic aspects of muscle wasting during critical illness. Curr. Opin. Clin. Nutr. Metab. Care.

[17] Welch C., Hassan-Smith Z.K., Greig C.A., Lord J.M., Jackson T.A. (2018). Acute Sarcopenia Secondary to Hospitalisation-An Emerging Condition Affecting Older Adults. Aging Dis..

[18] Liu W., Hu C., Zhao S. (2022). Sarcopenia and Mortality Risk of Patients with Sepsis: A Meta-Analysis. Int. J. Clin. Pract..

[19] Wahlen B.M., Mekkodathil A., Al-Thani H., El-Menyar A. (2020). Impact of sarcopenia in trauma and surgical patient population: A literature review. Asian J. Surg..

[20] Silva F.R.d.M., Souza A.S.R., Mello M.J.G.d., Santos P.A.A.d., Santos L.F.B.M.d., Fayh A.P.T., Sales J.T.d.O.L. (2026). Sarcopenia and early death in older patients with cancer: A secondary analysis of a prospective cohort. J. Geriatr. Oncol..

[21] Giakoustidis A., Papakonstantinou M., Chatzikomnitsa P., Gkaitatzi A.D., Bangeas P., Loufopoulos P.D., Louri E., Myriskou A., Moschos I., Antoniadis D. (2024). The Effects of Sarcopenia on Overall Survival and Postoperative Complications of Patients Undergoing Hepatic Resection for Primary or Metastatic Liver Cancer: A Systematic Review and Meta-Analysis. J. Clin. Med..

[22] Khan S., Benjamin J., Maiwall R., Tripathi H., Kapoor P.B., Shasthry V., Saluja V., Agrawal P., Thapar S., Kumar G. (2022). Sarcopenia is the independent predictor of mortality in critically ill patients with cirrhosis. J. Clin. Transl. Res..

[23] Skallerup A., Nygaard L., Olesen S.S., Køhler M., Vinter-Jensen L., Rasmussen H.H. (2018). The prevalence of sarcopenia is markedly increased in patients with intestinal failure and associates with several risk factors. Clin. Nutr..

[24] Fielding R.A., Vellas B., Evans W.J., Bhasin S., Morley J.E., Newman A.B., Abellan van Kan G., Andrieu S., Bauer J., Breuille D. (2011). Sarcopenia: An undiagnosed condition in older adults. Current consensus definition: Prevalence, etiology, and consequences. International working group on sarcopenia. J. Am. Med. Dir. Assoc..

[25] Studenski S.A., Peters K.W., Alley D.E., Cawthon P.M., McLean R.R., Harris T.B., Ferrucci L., Guralnik J.M., Fragala M.S., Kenny A.M. (2014). The FNIH sarcopenia project: Rationale, study description, conference recommendations, and final estimates. J. Gerontol. A Biol. Sci. Med. Sci..

[26] Chen L.-K., Woo J., Assantachai P., Auyeung T.-W., Chou M.-Y., Iijima K., Jang H.C., Kang L., Kim M., Kim S. (2020). Asian Working Group for Sarcopenia: 2019 Consensus Update on Sarcopenia Diagnosis and Treatment. J. Am. Med. Dir. Assoc..

[27] Kirk B., Cawthon P.M., Arai H., Ávila-Funes J.A., Barazzoni R., Bhasin S., Binder E.F., Bruyere O., Cederholm T., Chen L.-K. (2024). The Conceptual Definition of Sarcopenia: Delphi Consensus from the Global Leadership Initiative in Sarcopenia (GLIS). Age Ageing.

[28] Stevens R.D., Marshall S.A., Cornblath D.R., Hoke A., Needham D.M., de Jonghe B., Ali N.A., Sharshar T. (2009). A framework for diagnosing and classifying intensive care unit-acquired weakness. Crit. Care Med..

[29] Gade J., Beck A.M., Rønholt F., Andersen H.E., Munk T., Vinther A. (2020). Validation of the Danish SARC-F in Hospitalized, Geriatric Medical Patients. J. Nutr. Health Aging.

[30] Zoico E., Urbani S., Giani A., Fantin F., Gavras A., Batsis J.A., Micciolo R., Zamboni M. (2026). European Working Group on Sarcopenia in Older People Algorithm: Step-by-Step Relation With Length of Hospitalization. J. Am. Geriatr. Soc..

[31] Elmaleh D.M., Mohamed D.M.A. (2025). Sarcopenia and frailty as predictor of outcome in critically ill elderly patients with multiple comorbidities. Egypt. J. Intern. Med..

[32] Ibrahim K., Howson F.F.A., Culliford D.J., Sayer A.A., Roberts H.C. (2019). The feasibility of assessing frailty and sarcopenia in hospitalised older people: A comparison of commonly used tools. BMC Geriatr..

[33] Madrid N.M., Juárez R.R., Fernández-Jiménez R., Suarez A.V., Peláez P.G., Martín-Fontalba M.A., Medina B.F., Aguilar I.V., Campos M.d.M.A., Frapolli V.S. (2026). Body composition analysis and handgrip strength as predictors of 12-month mortality in hospitalized adults receiving parenteral nutrition: A descriptive cohort study. J. Parenter. Enter. Nutr..

[34] Hanna-Deschamps E., Herrmann F.R., Chirouzes D., Buratti L.C., Luthy C., Frangos E., Pautex S., Genton L., Zekry D., Graf C.E. (2026). Sarcopenia in Hospitalized Older Adults: A Cross-Sectional Study Comparing Diagnostic Thresholds and Handgrip Strength Measurement Tools. Geriatrics.

[35] Bertschi D., Kiss C.M., Beerli N., Kressig R.W. (2021). Sarcopenia in hospitalized geriatric patients: Insights into prevalence and associated parameters using new EWGSOP2 guidelines. Eur. J. Clin. Nutr..

[36] Kiss C.M., Bertschi D., Beerli N., Berres M., Kressig R.W., Fischer A.M. (2024). Calf circumference as a surrogate indicator for detecting low muscle mass in hospitalized geriatric patients. Aging Clin. Exp. Res..

[37] Darden N., Sharma S., Wu X., Mancini B., Karamchandani K., Bonavia A.S. (2023). Long-term clinical outcomes in critically ill patients with sepsis and pre-existing low muscle mass: A retrospective cohort study. BMC Anesthesiol..

[38] Kim D., Sun J.S., Lee Y.H., Lee J.H., Hong J., Lee J.-M. (2019). Comparative assessment of skeletal muscle mass using computerized tomography and bioelectrical impedance analysis in critically ill patients. Clin. Nutr..

[39] Paulus M.C., Melchers M., Kouw I.W.K., Vestering M., Viddeleer A.R., Zanten A.R.H. (2026). van Comparing bedside and CT-derived muscle mass assessment methodologies at intensive care unit admission: A critical step towards bedside detection of reduced muscle mass. Clin. Nutr..

[40] Kuchnia A., Earthman C., Teigen L., Cole A., Mourtzakis M., Paris M., Looijaard W., Weijs P., Oudemans-van Straaten H., Beilman G. (2017). Evaluation of Bioelectrical Impedance Analysis in Critically Ill Patients: Results of a Multicenter Prospective Study. J. Parenter. Enter. Nutr..

[41] Looijaard W.G.P.M., Stapel S.N., Dekker I.M., Rusticus H., Remmelzwaal S., Girbes A.R.J., Weijs P.J.M., Straaten H.M.O. (2020). Identifying critically ill patients with low muscle mass: Agreement between bioelectrical impedance analysis and computed tomography. Clin. Nutr..

[42] Ntoumenopoulos G., Hansell L., Parry S.M., Puthucheary Z. (2026). Diaphragm and Skeletal Muscle Changes During Intensive Care: Association With Patient Outcomes (DRASTIC): Observational Study. Physiother. Res. Int..

[43] Yanagi N., Koike T., Kamiya K., Hamazaki N., Nozaki K., Ichikawa T., Matsunaga A., Kuroiwa M., Arai M. (2021). Assessment of Sarcopenia in the Intensive Care Unit and 1-Year Mortality in Survivors of Critical Illness. Nutrients.

[44] Hernández-Socorro C.R., Saavedra P., López-Fernández J.C., Lübbe-Vazquez F., Ruiz-Santana S. (2021). Novel High-Quality Sonographic Methods to Diagnose Muscle Wasting in Long-Stay Critically Ill Patients: Shear Wave Elastography, Superb Microvascular Imaging and Contrast-Enhanced Ultrasound. Nutrients.

[45] Lopez-Ruiz A., Kashani K. (2020). Assessment of muscle mass in critically ill patients: Role of the sarcopenia index and images studies. Curr. Opin. Clin. Nutr. Metab. Care.

[46] Hermans G., Van Mechelen H., Clerckx B., Vanhullebusch T., Mesotten D., Wilmer A., Casaer M.P., Meersseman P., Debaveye Y., Van Cromphaut S. (2014). Acute Outcomes and 1-Year Mortality of Intensive Care Unit–acquired Weakness. A Cohort Study and Propensity-matched Analysis. Am. J. Respir. Crit. Care Med..

[47] Khalil A., Alamri R.A., Aljabri G.H., Shahat E.A., Almughamsi R.I., Almeshhen W.A. (2023). A Cross-Sectional Study of the Impact of ICU-Acquired Weakness: Prevalence, Associations, and Severity. Cureus.

[48] Liu G., Jiang S., Xie W., Liu X., Yang G., Lu W., Li H., Liu Z., Xiao W., Li Y. (2025). Biomarkers for sarcopenia, muscle mass, muscle strength, and physical performance: An umbrella review. J. Transl. Med..

[49] Papadopoulou S.K., Voulgaridou G., Kondyli F.S., Drakaki M., Sianidou K., Andrianopoulou R., Rodopaios N., Pritsa A. (2022). Nutritional and Nutrition-Related Biomarkers as Prognostic Factors of Sarcopenia, and Their Role in Disease Progression. Diseases.

[50] Wilkinson T.J., Baker L.A., Watson E.L., Smith A.C., Yates T. (2024). Diagnostic accuracy of a ‘sarcopenia index’ based on serum biomarkers creatinine and cystatin C in 458,702 UK Biobank participants. Clin. Nutr. ESPEN.

[51] Kashani K., Rosner M.H., Ostermann M. (2020). Creatinine: From physiology to clinical application. Eur. J. Intern. Med..

[52] Lian R., Liu Q., Jiang G., Zhang X., Tang H., Lu J., Yang M. (2024). Blood biomarkers for sarcopenia: A systematic review and meta-analysis of diagnostic test accuracy studies. Ageing Res. Rev..

[53] Voulgaridou G., Tyrovolas S., Detopoulou P., Tsoumana D., Drakaki M., Apostolou T., Chatziprodromidou I.P., Papandreou D., Giaginis C., Papadopoulou S.K. (2024). Diagnostic Criteria and Measurement Techniques of Sarcopenia: A Critical Evaluation of the Up-to-Date Evidence. Nutrients.

[54] Koga Y., Fujita M., Yagi T., Todani M., Nakahara T., Kawamura Y., Kaneda K., Oda Y., Tsuruta R. (2018). Early enteral nutrition is associated with reduced in-hospital mortality from sepsis in patients with sarcopenia. J. Crit. Care.

[55] Kondrup J., Rasmussen H.H., Hamberg O., Stanga Z., Ad Hoc ESPEN Working Group (2003). Nutritional risk screening (NRS 2002): A new method based on an analysis of controlled clinical trials. Clin. Nutr..

[56] Heyland D.K., Dhaliwal R., Jiang X., Day A.G. (2011). Identifying critically ill patients who benefit the most from nutrition therapy: The development and initial validation of a novel risk assessment tool. Crit. Care.

[57] Singer P., Blaser A.R., Berger M.M., Calder P.C., Casaer M., Hiesmayr M., Mayer K., Montejo-Gonzalez J.C., Pichard C., Preiser J.-C. (2023). ESPEN practical and partially revised guideline: Clinical nutrition in the intensive care unit. Clin. Nutr..

[58] Compher C., Bingham A.L., McCall M., Patel J., Rice T.W., Braunschweig C., McKeever L. (2022). Guidelines for the provision of nutrition support therapy in the adult critically ill patient: The American Society for Parenteral and Enteral Nutrition. JPEN J. Parenter. Enter. Nutr..

[59] da Silva J.S.V., Seres D.S., Sabino K., Adams S.C., Berdahl G.J., Citty S.W., Cober M.P., Evans D.C., Greaves J.R., Gura K.M. (2020). ASPEN Consensus Recommendations for Refeeding Syndrome. Nutr. Clin. Pract..

[60] Wunderle C., Gomes F., Schuetz P., Stumpf F., Austin P., Ballesteros-Pomar M.D., Cederholm T., Fletcher J., Laviano A., Norman K. (2023). ESPEN guideline on nutritional support for polymorbid medical inpatients. Clin. Nutr..

[61] Volkert D., Beck A.M., Cederholm T., Cruz-Jentoft A., Goisser S., Hooper L., Kiesswetter E., Maggio M., Raynaud-Simon A., Sieber C.C. (2019). ESPEN guideline on clinical nutrition and hydration in geriatrics. Clin. Nutr..

[62] Wischmeyer P.E., Bear D.E., Berger M.M., De Waele E., Gunst J., McClave S.A., Prado C.M., Puthucheary Z., Ridley E.J., Van den Berghe G. (2023). Personalized nutrition therapy in critical care: 10 expert recommendations. Crit. Care.

[63] Chapman M., Peake S.L., Bellomo R., Davies A., Deane A., Horowitz M., Hurford S., Lange K., Little L., TARGET Investigators, for the ANZICS Clinical Trials Group (2018). Energy-Dense versus Routine Enteral Nutrition in the Critically Ill. N. Engl. J. Med..

[64] Deutz N.E.P., Bauer J.M., Barazzoni R., Biolo G., Boirie Y., Bosy-Westphal A., Cederholm T., Cruz-Jentoft A., Krznariç Z., Nair K.S. (2014). Protein intake and exercise for optimal muscle function with aging: Recommendations from the ESPEN Expert Group. Clin. Nutr..

[65] Heyland D.K., Patel J., Compher C., Rice T.W., Bear D.E., Lee Z.-Y., González V.C., O’Reilly K., Regala R., Wedemire C. (2023). The effect of higher protein dosing in critically ill patients with high nutritional risk (EFFORT Protein): An international, multicentre, pragmatic, registry-based randomised trial. Lancet.

[66] Fetterplace K., Deane A.M., Tierney A., Beach L.J., Knight L.D., Presneill J., Rechnitzer T., Forsyth A., Gill B.M.T., Mourtzakis M. (2018). Targeted Full Energy and Protein Delivery in Critically Ill Patients: A Pilot Randomized Controlled Trial (FEED Trial). J. Parenter. Enter. Nutr..

[67] Verceles A.C., Serra M., Davis D., Alon G., Wells C.L., Parker E., Sorkin J., Bhatti W., Terrin M.L. (2023). Combining exercise, protein supplementation and electric stimulation to mitigate muscle wasting and improve outcomes for survivors of critical illness—The ExPrES study. Heart Lung J. Cardiopulm. Acute Care.

[68] Burd N.A., Gorissen S.H., van Loon L.J.C. (2013). Anabolic resistance of muscle protein synthesis with aging. Exerc. Sport. Sci. Rev..

[69] Ely I.A., Phillips B.E., Smith K., Wilkinson D.J., Piasecki M., Breen L., Larsen M.S., Atherton P.J. (2023). A focus on leucine in the nutritional regulation of human skeletal muscle metabolism in ageing, exercise and unloading states. Clin. Nutr..

[70] García-Alonso A., Sánchez-González J.L., Navarro-López V., Méndez-Sánchez R., Polo-Ferrero L. (2025). The Role of HMB Supplementation in Enhancing the Effects of Resistance Training in Older Adults: A Systematic Review and Meta-Analysis on Muscle Quality, Body Composition, and Physical Function. Nutrients.

[71] Wulandari Y., Aditianingsih D., Sunardi D., Prasetyo M., Madjid A.S., Nusdwinuringtyas N., Sutanto L.B., Hardy G. (2026). High leucine branched-chain amino acids supplementation ameliorates quadriceps femoris muscle thickness reduction and attenuates interleukin-6 in critically ill patients: A randomised controlled trial. Clin. Nutr. ESPEN.

[72] Wandrag L., Brett S.J., Frost G.S., To M., Loubo E.A., Jackson N.C., Umpleby A.M., Bountziouka V., Hickson M. (2019). Leucine-enriched essential amino acid supplementation in mechanically ventilated trauma patients: A feasibility study. Trials.

[73] Wu T., Wei Y., Xiong J., Wu J., Lin X., Zhuang Y., Luo C., Xu M., Chen X., Lin Z. (2025). Resistance training and β-hydroxy-β-methylbutyrate for functional recovery in critical illness: A multicenter 2 × 2 factorial randomized trial. Crit. Care.

[74] Vegas-Aguilar I., Fernández-Jiménez R., Cornejo-Pareja I., Amaya-Campos M.D.M., Guirado-Peláez P., Montero-Madrid N., Vidal-Suarez Á., Martín-Fontalba M.A., Simon-Frapolli V., Tinahones F.J. (2025). Adherence to β-hydroxy-β-methylbutyrate-Enriched Oral Nutritional Supplements Enhances Survival and Nutritional Recovery in Malnourished Outpatients: Prognostic Insights. Nutrients.

[75] Gao X., Liu S., Zhang Y., Xia Y., Liu S., Qi X., Xiao Y., Zhu M., Sun G., Gao T. (2025). Effect of vitamin D supplementation on skeletal muscle function and quality of life in patients with chronic intestinal failure: A randomized controlled trial. J. Transl. Med..

[76] Rondanelli M., Cereda E., Klersy C., Faliva M.A., Peroni G., Nichetti M., Gasparri C., Iannello G., Spadaccini D., Infantino V. (2020). Improving rehabilitation in sarcopenia: A randomized-controlled trial utilizing a muscle-targeted food for special medical purposes. J. Cachexia Sarcopenia Muscle.

[77] Calder P.C. (2020). Nutrition, immunity and COVID-19. BMJ Nutr. Prev. Health.

[78] Hossain T., Phillips B.E., Doleman B., Lund J.N., Williams J.P. (2020). A double-blind randomized controlled trial of the effects of eicosapentaenoic acid supplementation on muscle inflammation and physical function in patients undergoing colorectal cancer resection. Clin. Nutr..

[79] Therdyothin A., Prokopidis K., Galli F., Witard O.C., Isanejad M. (2025). The effects of omega-3 polyunsaturated fatty acids on muscle and whole-body protein synthesis: A systematic review and meta-analysis. Nutr. Rev..

[80] Heyland D., Muscedere J., Wischmeyer P.E., Cook D., Jones G., Albert M., Elke G., Berger M.M., Day A.G. (2013). Canadian Critical Care Trials Group. A randomized trial of glutamine and antioxidants in critically ill patients. N. Engl. J. Med..

[81] Reignier J., Boisramé-Helms J., Brisard L., Lascarrou J.-B., Ait Hssain A., Anguel N., Argaud L., Asehnoune K., Asfar P., Bellec F. (2018). Enteral versus parenteral early nutrition in ventilated adults with shock: A randomised, controlled, multicentre, open-label, parallel-group study (NUTRIREA-2). Lancet.

[82] Reignier J., Plantefeve G., Mira J.-P., Argaud L., Asfar P., Aissaoui N., Badie J., Botoc N.-V., Brisard L., Bui H.-N. (2023). Low versus standard calorie and protein feeding in ventilated adults with shock: A randomised, controlled, multicentre, open-label, parallel-group trial (NUTRIREA-3). Lancet Respir. Med..

[83] Casaer M.P., Mesotten D., Hermans G., Wouters P.J., Schetz M., Meyfroidt G., Van Cromphaut S., Ingels C., Meersseman P., Muller J. (2011). Early versus late parenteral nutrition in critically ill adults. N. Engl. J. Med..

[84] Lin Y.-R., Chen P.-C., Li W.-T., Huang M.-H., Huang S.-F., Wang C.-J., Chien Y.-W., Kao A.-W., Shan Y.-S. (2025). The relationship between caloric intake and clinical outcomes in critically ill patients: A retrospective study. Clin. Nutr. ESPEN.

[85] Moriwaki M., Wakabayashi H., Sakata K., Domen K. (2019). The Effect of Branched Chain Amino Acids-Enriched Nutritional Supplements on Activities of Daily Living and Muscle Mass in Inpatients with Gait Impairments: A Randomized Controlled Trial. J. Nutr. Health Aging.

[86] Deutz N.E., Matheson E.M., Matarese L.E., Luo M., Baggs G.E., Nelson J.L., Hegazi R.A., Tappenden K.A., Ziegler T.R. (2016). NOURISH Study Group. Readmission and mortality in malnourished, older, hospitalized adults treated with a specialized oral nutritional supplement: A randomized clinical trial. Clin. Nutr..

[87] Cioffi I., Ponzo V., Pellegrini M., Evangelista A., Bioletto F., Ciccone G., Pasanisi F., Ghigo E., Bo S. (2021). The incidence of the refeeding syndrome. A systematic review and meta-analyses of literature. Clin. Nutr..

[88] Liu C., He L., Zhang J.H., He J., Tian L., Zheng X. (2024). Impact of high-protein enteral nutrition on muscle preservation in mechanically ventilated patients with severe pneumonia: A randomized controlled trial. J. Health Popul. Nutr..

[89] Seifi N., Rezvani R., Sedaghat A., Nematy M., Khadem-Rezaiyan M., Safarian M. (2022). The effects of synbiotic supplementation on enteral feeding tolerance, protein homeostasis, and muscle wasting of critically ill adult patients: A randomized controlled trial. Trials.

[90] Oke S.M., Rye B., Malietzis G., Baldwin-Cleland R., Bottle A., Gabe S.M., Lung P.F.C. (2020). Survival and CT defined sarcopenia in patients with intestinal failure on home parenteral support. Clin. Nutr..

[91] Dresen E., Weißbrich C., Fimmers R., Putensen C., Stehle P. (2021). Medical high-protein nutrition therapy and loss of muscle mass in adult ICU patients: A randomized controlled trial. Clin. Nutr..

[92] Dickerson R.N., Buckley C.T. (2021). Impact of Propofol Sedation upon Caloric Overfeeding and Protein Inadequacy in Critically Ill Patients Receiving Nutrition Support. Pharmacy.

[93] Wischmeyer P.E., Hasselmann M., Kummerlen C., Kozar R., Kutsogiannis D.J., Karvellas C.J., Besecker B., Evans D.K., Preiser J.-C., Gramlich L. (2017). A randomized trial of supplemental parenteral nutrition in underweight and overweight critically ill patients: The TOP-UP pilot trial. Crit. Care.

[94] Stoian M., Andone A., Bândilă S.R., Onișor D., Babă D.-F., Niculescu R., Stoian A., Azamfirei L. (2025). Personalized Nutrition Strategies for Patients in the Intensive Care Unit: A Narrative Review on the Future of Critical Care Nutrition. Nutrients.

[95] Fazzini B., Märkl T., Costas C., Blobner M., Schaller S.J., Prowle J., Puthucheary Z., Wackerhage H. (2023). The rate and assessment of muscle wasting during critical illness: A systematic review and meta-analysis. Crit. Care.

